# Use of medications with pharmacogenomic guidelines and adverse outcomes in hospitalised older patients: a retrospective cross-sectional study

**DOI:** 10.1038/s41397-026-00396-3

**Published:** 2026-01-28

**Authors:** Victoria David, Ciarán D. McInerney, Justine Tomlinson, Eleanor Bryant, Gurdeep S. Sagoo, V-Lin Cheong, Heather Smith, Beth Fylan, Marcus Rattray

**Affiliations:** 1 School of Pharmacy, Optometry and Medical Sciences, Faculty of Health and Social Care, University of Bradford, Bradford, UK; 2 Medicines Management and Pharmacy Services, Leeds Teaching Hospitals NHS Trust, Leeds, UK; 3 Wolfson Centre for Applied Health Research, Bradford, UK; 4 NIHR Yorkshire & Humber Patient Safety Research Collaboration, Bradford, UK; 5 Academic Unit of Primary Medical Care, University of Sheffield, Sheffield, UK; 6 Department of Psychology, School of Law and Social Sciences and, Faculty of Management, Sciences and Engineering, University of Bradford, Bradford, UK; 7 Population Health Sciences Institute, Faculty of Medical Sciences, Newcastle University, Newcastle, UK; 8 School of Healthcare, University of Leeds, Leeds, UK; 9 NHS West Yorkshire Integrated Care Board, wakefield, UK; 10 Yorkshire Quality and Safety Research Group, Bradford Institute for Health Research, Bradford, Yorkshire UK

**Keywords:** Health services, Geriatrics

## Abstract

This study aimed to assess the prevalence of the use of medications with pharmacogenomic guidelines upon hospital admission in patients aged 65 and over and evaluate its association with adverse outcomes, including length of stay, unplanned admissions, and repeat hospital admissions. A retrospective cross-sectional study was conducted using hospital admissions data from 2018–2019 in one NHS hospital trust in England, focusing on patients aged 65 and over. The usage of medications with pharmacogenomic guidelines was examined, and comparisons were made between their prevalence in unplanned and planned admissions. Multivariable models assessed whether the use of medications with pharmacogenomic guidelines were associated with adverse outcomes, considering frailty status. Analysis of 59,973 admissions revealed 67 pharmacogenomics medicines as per the Clinical Pharmacogenetics Implementation Consortium (CPIC) guidelines, with 11 classified as high-risk among 1438 unique medicines identified from 560,179 recorded medications. Notably, unplanned admissions exhibited a higher prevalence of medications with pharmacogenomic guidelines (84% versus 64%, p < 0.001) compared to planned admissions. The models demonstrated the usage of these medications was associated with adverse outcomes (length of stay in hospital, unplanned admission and repeat hospital admission) with substantial evidence (Delta_AICc < 2) particularly in patients with high frailty status. This study highlights the association between medications with pharmacogenomic guidelines and adverse outcomes, particularly among patients with high frailty. The findings emphasise the importance of integrating pharmacogenomic-guided care into the management of older individuals with frailty to mitigate adverse outcomes and enhance medication safety.

## Introduction

Medicines optimisation plays a crucial role in reducing adverse drug reactions (ADRs), which are harmful and unintended responses to medicines [1]. Most ADRs are theoretically preventable; however, they continue to play a significant role in hospital admissions and repeat hospitalisations, being responsible for 4 - 6.5% of unplanned hospital admissions [2–4] and 16% or more of repeat hospitalisation [5–7]. Moreover, patients experiencing ADRs tend to have a longer hospital stay compared to those without ADRs [8]. A recent UK study identified age, co-morbidities and number of prescribed medicines as significant factors associated with ADRs [4]. Older people (aged 65 and over) are particularly vulnerable to ADRs, with twice the likelihood of medicine-related hospital admissions than their younger counterparts [9, 10] and up to 30% of hospital admissions in older patients attributable to medicines [11]. This heightened vulnerability is associated with frailty, a state of diminished ability to recover from stressor events [12] particularly among hospitalised patients [13–15], as well as multimorbidity and inappropriate polypharmacy [16–18]. Given that age is a non-modifiable risk factor and polypharmacy is often necessary due to multimorbidity, it is important to identify patients at an elevated risk of ADRs to inform medicines selection and dosage optimisation.

Pharmacogenomics (PGx), the study of genetic variation and their effects on drug response, offers a promising approach to enhance prescribing safety and efficacy, thus reducing ADRs [4]. It is estimated that more than 95% of the population have at least one gene variant that would affect their response to at least one medicine [19–21]. There are numerous clinically relevant interactions between drugs and gene variants that have been identified. Currently, the Clinical Pharmacogenetics Implementation Consortium (CPIC) has identified 111 gene-drug pairs at level A or B, where drug-gene interactions have sufficient evidence to warrant at least one prescribing action recommendation, such as dose adjustment or alternate drug [22]. The extent of prescribing of medicines with actionable PGx associations is considerable. For instance, a recent UK study showed that about 19.1%21.1% of new prescriptions in primary care had a PGx association [23]. Furthermore, a prospective study of 1000 adults demonstrated that 30% of ADRs at hospital admission were caused by at least one drug with a PGx association [24]. Over a 20-year period, almost 90% of older patients in English primary care were exposed to at least one medication with PGx guidelines, with exposure to such medicines markedly increasing with age [25].

Although pharmacogenomic tests are not yet routinely used across healthcare systems, there is growing evidence supporting the positive impact of PGx-guided care on reducing adverse outcomes [23, 25]. To date, no studies have examined the extent to which medicines with actionable drug-gene interactions are associated with adverse outcomes in older people. Consequently, this study aims to use hospital records to establish the prevalence of medications with PGx guidelines among hospitalised patients and determine whether the use of these medications is associated with a patient’s length of stay in hospital, unplanned admission and repeat hospital admission. These findings can support decisions about implementation of PGx-guided care in health systems.

## Methods

### Study design and participants

This study employed a retrospective cross-sectional design to analyse hospital admissions data for all patients aged 65 and over in 2018 and 2019 to one National Health Service (NHS) Teaching Hospital Trust in England. The admissions dataset was de-identified record-level data obtained from the Leeds Teaching Hospitals NHS Trust and approved by the Data Access Committee in the Leeds Teaching Hospitals NHS Trust. The study was reviewed and approved by the University of Bradford Ethics Committee Chair (IRAS190) and submitted to the Health Research Authority who granted study approval (21/HRA/3937).

In this study, medications with PGx guidelines were defined as those with actionable drug-gene interactions of high-level evidence (CPIC Level A and B) prioritised by CPIC. High risk medicines were identified as medicines that cause ADRs implicated in hospitalisation, as listed by Pirmohamed et al. [2].

Frailty status was assessed using the Hospital Frailty Risk Score (HFRS), a validated tool based on ICD-10 codes [26]. The HFRS was calculated using diagnosis codes recorded during inpatient episodes within the two years prior to the discharge date. Patients were categorised as having low, intermediate, or high frailty based on their HFRS score. Where the score could not be calculated due to the absence of prior hospitalisation data, patients were recorded as “frailty not calculated”.

To investigate the potential relationship between medications with PGx guidelines and adverse outcomes (prolonged hospital stay, unplanned admission and repeat hospital admission), predictive models were developed. The outcomes were prolonged hospital stay, unplanned admission, and repeat hospital admission within a 24-month timeframe. Key predictor variables included age, sex, ethnicity, frailty status, total number of medications, and the number of medications with PGx guidelines prescribed. Due to limitations in coding data, unplanned admissions were used as a proxy for potential ADR-related admissions, rather than direct identification of ADRs.

### Analysis

The dataset was cleaned and analysed using R version 4.1.1 (R Core Team 2021). To investigate the prevalence of medications with PGx guidelines recorded upon hospital admission, each medicine was ranked by frequency of occurrence. Descriptive statistics were used to explore the composition of the admissions dataset in terms patient medicine use and outcome.

A Mann-Whitney U test was applied to compare medians (length of stay, number of hospitalisations, total number of medications, those without and those with PGx guidelines) between planned and unplanned admissions. Chi-squared tests were used when comparing the type of admission (unplanned or planned) and the number of medications with PGx guidelines observed in the dataset, with statistical significance indicated by p < 0.05.

The admissions dataset contained three prediction outcomes selected as adverse outcomes: prolonged hospital stay (discrete variable), the occurrence of an unplanned admission (categorical variable) and the occurrence of a repeat hospital admission (categorical variable). Selection of potential predictors for these outcomes relied on the all-subset variable selection method, which identifies the best-performing multivariable regression model from all the possible models using the available predictor variables [27]. The residual sum of squares was measured for each subset and the one with the lowest residual sum of squares was selected.

The selected variables were also evaluated for collinearity, a scenario where predictor variables are highly correlated and therefore unable to independently predict the outcome variable [28]. Collinearity is reported as variance inflation factor (VIF), with a value exceeding 10 indicating the presence of multicollinearity [29].

Multivariable prediction models were developed to investigate the relationship between predictor variables and adverse outcomes, generating equations containing predictor variables and their exponentiated coefficients. The coefficients and the constant in this model represent the unique contribution of each predictor variable to the outcome while considering the effects of all the predictor variables in the model. The magnitude and direction of the coefficients may not directly correspond to the relationship between each predictor variable and the outcome variable when examined in separately.

The study employed a comparative assessment of multivariable prediction models to discern the importance of using medications with PGx guidelines in predicting adverse outcomes. In these models, the number of medications with PGx guidelines documented in the patient record was either included or excluded. The performance of models with and without the PGx variable were compared using corrected Akaike’s Information Criteria (AICc), a statistic used to assist in the selection of a statistical model from a set of candidate models. The AICc estimates the amount of information lost by a proposed model. In comparing two models, the one presenting a lower AICc score means less information loss and a better model fit to the admission dataset. When the AIC score of one multivariable prediction model exceeds that of another by more than two units, the latter is regarded as superior [30, 31]. The differences in AICc scores between models are presented in the Delta_AICc column. The cumulative model weight (Cum.Wt) is the sum of the AICc (corrected AIC) weights reported in the results to reflect the likelihood of each model being the best fit for the data. This methodological approach aids in the robust evaluation of the role of medications with PGx guidelines in predicting adverse patient outcomes.

## Results

### Patient characteristics and pharmacogenomic-associated medicines use

From the 532,711 instances of medicines use documented in the dataset, a total of 1432 unique medicines were identified. Of these, 63 were classified as medications with PGx guidelines according to CPIC and 12 were identified as high risk (Appendix Table A1). The five most commonly prescribed medications with PGx guidelines were lansoprazole, atorvastatin, simvastatin, codeine and omeprazole (Table 1).

**Table 1:** Summary of CPIC Level A–B Medicines: Prescription Prevalence, Pharmacogenomic Risk, and Gene Variant Frequencies.

PGx medicine	Number of unique patients prescribed CPIC A-B medications	Proportion of patients prescribed CPIC A-B medications (%)	High risk	Genes contributing to the recommendation	*Frequency of people with gene variants requiring atypical dosage guidance
lansoprazole	14712	32.78	no	CYP2C19	4.4%. increased dose
atorvastatin	14256	31.76	no	SLCO1B1	not available in biobank.
codeine	9999	22.28	yes	CYP2D6	6.01%. alternate drug
simvastatin	9078	20.23	no	SLCO1B1	22.9%. decreased dose
omeprazole	8162	18.19	no	CYP2C19	5.56%. increased dose
clopidogrel	5351	11.92	yes	CYP2C19	27.9%. alternate drug
warfarin	3962	8.83	yes	CYP4F2, VKORC1, CYP2C19	48.06%. decreased dose
amitriptyline	3508	7.82	yes	CYP2D6, CYP2C19	37.9%. alternate drug; 23.9%. decreased dose
ibuprofen	3027	6.74	yes	CYP2C9	14.3%. decreased dose
allopurinol	2980	6.64	no	HLA-B	not available in biobank.
sertraline	2285	5.09	yes	CYP2C19	2.16%. decreased dose
citalopram	1968	4.39	yes	CYP2C19	4.8%. alternate drug; 0.3%. decreased dose
tramadol	1868	4.16	yes	CYP2D6	not available in biobank.
quinine	1793	4.00	no	G6PD	not available in biobank.
nitrofurantoin	1212	2.70	no	G6PD	not available in biobank.
rosuvastatin	935	2.08	no	ABCG2	not available in biobank.
ondansetron	930	2.07	no	CYP2D6	not available in biobank.
piroxicam	756	1.68	no	CYP2C9	6.8%. alternate drug
pantoprazole	627	1.40	no	CYP2C19	4.4%. increased dose
venlafaxine	427	0.95	yes	CYP2D6	not available in biobank.
glimepiride	402	0.90	no	G6PD	not available in biobank.
risperidone	396	0.88	no	CYP2D6	not available in biobank.
ciprofloxacin	349	0.78	no	G6PD	not available in biobank.
valproate	339	0.76	no	OTC	not available in biobank.
carbamazepine	328	0.73	no	HLA-A, HLA-B	not available in biobank.
mesalazine	302	0.67	no	G6PD	not available in biobank.
chloramphenicol	250	0.56	no	G6PD	not available in biobank.
paroxetine	237	0.53	yes	CYP2D6	5.6%. alternate drug
sulfasalazine	215	0.48	no	G6PD	not available in biobank.
mycophenolate	201	0.45	no	HPRT1	not available in biobank.
tacrolimus	196	0.44	no	CYP3A5	not available in biobank.
azathioprine	193	0.43	no	NUDT15, TPMT	11.2%. decreased dose; 0.7%. alternate drug
phenytoin	159	0.35	no	CYP2C9, HLA-B	16.5%. decreased dose
celecoxib	134	0.30	no	CYP2C9	14.4%. decreased dose
meloxicam	132	0.29	no	CYP2C9	13%. decreased dose; 1.9%. alternate drug
tamoxifen	107	0.24	no	CYP2D6	48.3%. alternate drug
nortriptyline	97	0.22	no	CYP2D6	33.5%. decreased dose; 6.1%. alternate drug
hydralazine	94	0.21	no	NAT2	not available in biobank.
escitalopram	86	0.19	yes	CYP2C19	3.4%. alternate drug; 1.8%. decreased dose
aripiprazole	70	0.16	no	CYP2D6	not available in biobank.
clomipramine	54	0.12	no	CYP2D6, CYP2C19	42%. alternate drug; 21.6%. decreased dose
gentamicin	42	0.09	no	MT-RNR1	not available in biobank.
capecitabine	38	0.08	no	DPYD	not available in biobank.
fluorouracil	35	0.08	no	DPYD	6.8%. decreased dose
imipramine	33	0.07	no	CYP2D6, CYP2C19	39%. alternate drug; 22.9%. decreased dose
dapsone	30	0.07	no	G6PD	not available in biobank.
doxepin	24	0.05	no	CYP2D6	31.4%. decreased dose
mercaptopurine	18	0.04	no	NUDT15, TPMT	not available in biobank.
glipizide	13	0.03	no	G6PD	not available in biobank.
acenocoumarol	12	0.03	no	CYP4F2	29.4%. alternate drug
methadone	12	0.03	no	CYP2B6	not available in biobank.
tetrabenazine	12	0.03	no	CYP2D6	not available in biobank.
trimipramine	9	0.02	no	CYP2C19, CYP2D6	34%. alternate drug; 18%. decreased dose
voriconazole	9	0.02	no	CYP2C19	not available in biobank.
oxcarbazepine	6	0.01	no	HLA-B	not available in biobank.
abacavir	5	0.01	no	HLA-B	not available in biobank.
efavirenz	5	0.01	no	CYP2B6	not available in biobank.
glibenclamide	5	0.01	no	G6PD	not available in biobank.
amikacin	2	0.00	no	MT-RNR1	not available in biobank.
fluvoxamine	1	0.00	no	CYP2D6	54.5%. decreased dose
pimozide	1	0.00	no	CYP2D6	not available in biobank.
vortioxetine	1	0.00	no	CYP2D6	not available in biobank.

^*^Frequency of patients with gene variants requiring atypical dosage guidance is based on McInnes et al. [41], derived from 36,226 UK Biobank participants.

The admissions dataset encompassed 59,973 hospital admissions for 35,390 unique patients with at least one medicine recorded upon admission containing key predictor (independent variables) and outcome variables for this analysis, including characteristics, medicines recorded upon hospital admission and various clinical outcomes (Table 2). Table 2 further subdivides this patient population into those with medicines recorded on hospital admission, categorising them based on whether their hospital admission was planned or unplanned.

**Table 2:** Characteristics of hospital admissions dataset (2018–2019) in patients aged 65 and over.

	Admissions with medications	Unplanned Admissions with medications	Planned Admissions with medications	Chi-square (χ^2^) and Mann-Whitney U test: unplanned with medications vs planned with medications	p-value
**Number of admissions (% of admissions with medicines)**	59973 (100)	49574 (82.66)	10399 (17.34)		
**Median length of stay, days (IQR**)	5.00 (2, 13)	6.00 (2, 14)	4.00 (2, 8)	25590	<0.001
**Median number of hospitalisations (IQR)**	3.00 (2, 6)	4.00 (2, 7)	2.00 (1, 4)	25590	<0.001
**Number admitted due to ‘unspecified adverse effect of drug or medicament’ (T887) (% of total admissions)**	18 (0.03)	18 (0.04)	0	−4.24	<0.001
**Number died as inpatient (% of number of admissions)**	4013 (6.7)	3827 (7.7)	186 (1.8)	33.54	<0.001
**Total number of medicines (median (IQR))**	9.00 (6, 12)	9.00 (6, 13)	7.00 (2, 8)	25590	<0.001
**Non-PGx medicines (median (IQR))**	7.00 (4, 10)	7.00 (4, 10)	5.00 (3, 8)	25590	<0.001
**PGx medicines (median (IQR))**	2.00 (1, 3)	2.00 (1, 3)	2.00 (1, 3)	25590	<0.001
**Age Band on Admission (% of total admissions of all age bands)**				2968.2	<0.001
[65–74]	21246 (35.4)	15562 (31.4)	5684 (54.7)		
[75–84]	22556 (37.6)	18687 (37.7)	3869 (37.2)		
[85 + ]	16171 (27.0)	15325 (30.9)	846 (8.1)		
**Sex (% male)**	28901 (48.2)	23150 (46.7)	5751 (55.3)	254.62	<0.001
**Number of admissions by frailty status (% of total admissions of all frailty bands)**				6475.8	<0.001
High	16748 (27.9)	16243 (32.8)	505 (4.9)		
Intermediate	20087 (33.5)	17692 (35.7)	2395 (23.0)		
Low	22339 (37.2)	15057 (30.4)	7282 (70.0)		
Not calculated	799 (1.3)	582 (1.2)	217 (2.1)		
**Broad Ethnic Group (% of total admissions of all ethnicities)**				424.62	<0.001
Asian	1949 (3.2)	1661 (3.4)	288 (2.8)		
Black	621 (1.0)	548 (1.1)	73 (0.7)		
Mixed	114 (0.2)	97 (0.2)	17 (0.2)		
Not Stated	1457 (2.4)	918 (1.9)	539 (5.2)		
Other	294 (0.5)	231 (0.5)	63 (0.6)		
White	55538 (92.6)	46119 (93.0)	9419 (90.6)		

Unplanned hospital admissions in patients over 65 were more likely to involve females, older individuals, patients living with frailty and non-white patients (Table 2). Compared to planned admissions, unplanned admission had a longer hospital stay (6 days versus 4 days, p < 0.001), twice the number of hospital admissions (4 versus 2, p < 0.001) and were more likely to be due to an ADR. Additionally, inpatient mortality was higher in unplanned admissions (7.7% versus 1.8%, p < 0.001) (Table 2).

**Fig. 1:**
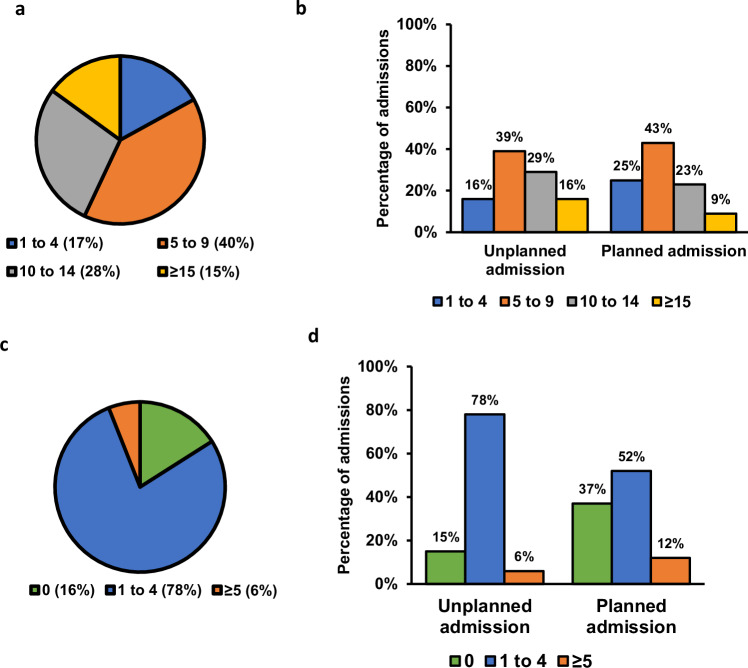
Distribution of medications per hospital admission. **a** Total medications per admission. **b** Total medications per admission by unplanned vs. planned admissions (χ²(2, N = 59,973) = 770.021, p < 0.001). Y-axis shows percentage of admissions**. c** Medications with PGx guidelines per admission. **d** Medications with PGx guidelines per admission by unplanned vs. planned admissions (χ²(2, N = 59,973) = 93.459, p < 0.001). Y-axis shows percentage of admissions. Coloured bars indicate the proportion of admissions in each category.

Figure 1 shows the number of medicines (Fig. 1a) or pharmacogenomic medicines (Fig. 1c) taken by patients upon hospital admission and provides a visual comparison of the differences between patients with planned and unplanned admission in relation to the number of medicines used (Fig. 1b) and the number of pharmacogenomic medicines used (Fig. 1d). Patients over 65 with unplanned hospital admissions were found to be on more medicines (9 versus 7, p < 0.001) (Fig. 1b). Data indicated that, while a similar proportion of patients in both planned and unplanned admissions were prescribed at least one medication with PGx guidelines, patients with unplanned admissions were taking a higher number of PGx medications per admission (84% versus 64%, p < 0.001) compared to patients with planned admissions (Fig. 1d).

### Testing the association between use of medications with pgx guidelines and adverse outcomes in patients aged 65 and over

The association between the use of medications with PGx guidelines and adverse outcomes in patients aged 65 and over was investigated. A higher prevalence of medications with PGx guidelines was observed among patients with unplanned admissions compared to those with planned admissions (Fig. 1d). Consequently, we tested if the use of medications with PGx guidelines was associated with an increased predicted risk of adverse outcomes, namely unplanned admissions, length of stay in hospital and repeat hospital admission.

To ensure the validity of the multivariable prediction models, the predictor variables (age, gender, ethnicity, frailty status, number of medicines used, number of medications with PGx guidelines used) were evaluated for collinearity (Appendix Table A2). The collinearity test reported a low Variance Inflation Factor (VIF), a low standard error and a tolerance close to 1 which indicated minimal multicollinearity, and that all the variables could be included in model construction. The all-subset variable selection method was then employed to determine which predictors from the candidate set were optimal for predicting for the outcomes of interest (Appendix Table A3). The residual sum of squares is measured for each subset and the best subset of variables with the lowest residual sum of squares was selected. The subset that contained all the predictor variables available had the lowest residual sum of squares (Appendix Table A3).

Subsequently, multivariable prediction models were developed using the admissions dataset, incorporating the number of medications with PGx guidelines used and other predictor variables (Appendix Table A4). Frailty status was assessed using the HFRS, categorising patients as low, intermediate, or high frailty based on diagnosis codes from prior hospitalisations. The models provided insights into the relative contribution of each variable to the outcomes. Based on the coefficients in the prediction model equations (Appendix Table A4), higher frailty categories were associated with substantially greater risk; for example, in the unplanned admission model, high frailty had a coefficient of 9.64 and intermediate frailty 2.84, compared to 1.37 for ‘frailty not calculated’. This confirms that frailty was consistently the highest weighted predictor of poorer patient outcomes, which led to further analysis stratified by frailty status (Appendix Table A5).

The models show that each additional medication with PGx guidelines, when other variables are held constant, increases the length of hospital stay, the likelihood of unplanned admission and the risk of hospital repeat hospital admission. To determine the best-performing multivariable prediction models, we compared the AICc of models incorporating PGx versus those excluding it (Table 3). For all outcomes of interest, the model incorporating the number of medications with PGx guidelines was the best-fit for predicting length of stay (AICc = 989,779.1; cumulative weight = 100%), unplanned admission (AICc = 46,524.63; cumulative weight = 100%) and repeat hospital admission (after planned admission: AICc = 18,028.37; cumulative weight = 65%; after an unplanned admission: AICc = 153,840.2; cumulative weight = 98%).

**Table 3:** Multivariable prediction model Selection based on the corrected Akaike Information Criteria (AICc).

Length of stay					
	K	AICc	Delta_AICc	AICc_Wt	Cum.Wt
Model with PGx	16	989779.10	0.00	1.00	1.00
Model without PGx	15	989846.40	67.30	0.00	0.00
Model without the total number of medicines	15	989904.65	125.55	0.00	0.00
**Unplanned admission**					
	K	AICc	Delta_AICc	AICc_Wt	Cum.Wt
Model with PGx	15	46524.63	0.00	1.00	1.00
Model without PGx	14	46554.30	29.67	0.00	0.00
Model without the total number of medicines	14	46712.83	188.20	0.00	0.00
**Repeat hospitalisation after a planned admission**					
	K	AICc	Delta_AICc	AICc_Wt	Cum.Wt
Model without PGx	14	18028.37	0.00	0.65	0.65
Model with PGx	15	18029.58	1.21	0.35	1.00
Model without the total number of medicines	14	18059.69	31.32	0.00	1.00
**Repeat hospitalisation after unplanned admission**					
	K	AICc	Delta_AICc	AICc_Wt	Cum.Wt
Model with PGx	15	153840.2	0.00	0.98	0.98
Model without PGx	14	153848.2	7.96	0.02	1.00
Model without the total number of medicines	14	155016.5	1176.26	0.00	1.00

Delta_AICc = the difference in AIC score between the best model and the model being compared.

K = is the number of independent variables.

In high frailty patients, the best-fit model for length of stay included the number of medications with PGx guidelines, evidenced by the lowest AICc score and carrying 100% of the cumulative model weight (Table 4). For intermediate and low frailty patients, the models excluding PGx were comparable in predictive performance to those including PGx, with neither model demonstrating significant superiority (Delta_AICc <2) in predicting the outcome variable (Table 4).

**Table 4:** Multivariable prediction model Selection based on the corrected Akaike Information Criteria (AICc) stratified by frailty status.

Length of stay					
High Frailty					
	K	AICc	Delta_AICc	AICc_Wt	Cum.Wt
Model with PGx	13	435074.7	0.00	1.00	1.00
Model without PGx	12	435170.1	95.37	0.00	0.00
Model without the total number of medicines	12	435194.8	120.13	0.00	0.00
**Intermediate Frailty**					
	K	AICc	Delta_AICc	AICc_Wt	Cum.Wt
Model without PGx	12	344083.9	0.00	0.65	0.65
Model with PGx	13	344085.2	1.25	0.35	1.00
Model without the total number of medicines	12	344196.0	112.08	0.00	1.00
**Low Frailty**					
	K	AICc	Delta_AICc	AICc_Wt	Cum.Wt
Model without PGx	12	207186.5	0.00	0.73	0.73
Model with PGx	13	207188.5	1.99	0.27	1.00
Model without the total number of medicines	12	207248.0	61.55	0.00	0.00
**Unplanned admission**					
**High Frailty**					
	K	AICc	Delta_AICc	AICc_Wt	Cum_Wt
Model with PGx	12	4260.74	0.00	0.79	0.79
Model without the total number of medicines	11	4263.63	2.89	0.19	0.98
Model without PGx	11	4267.88	7.14	0.02	1.00
**Intermediate Frailty**					
	K	AICc	Delta_AICc	AICc_Wt	Cum_Wt
Model with PGx	12	14036.32	0.00	1.00	1.00
Model without PGx	11	14048.88	12.55	0.00	1.00
Model without the total number of medicines	11	14093.21	56.88	0.00	1.00
**Low Frailty**					
	K	AICc	Delta_AICc	AICc_Wt	Cum_Wt
Model with PGx	12	27112.32	0.00	1.00	1.00
Model without PGx	11	27125.48	13.16	0.00	1.00
Model without the total number of medicines	11	27249.29	136.97	0	1.00
**Repeat hospitalisation after an unplanned admission**					
**High Frailty**					
	K	AICc	Delta_AICc	AICc_Wt	Cum_Wt
Model with PGx	12	59273.39	0.00	1	1.00
Model without PGx	11	59287.38	13.99	0	1.00
Model without the total number of medicines	11	59612.51	339.12	0	1.00
**Intermediate Frailty**					
	K	AICc	Delta_AICc	AICc_Wt	Cum_Wt
Model without PGx	11	54438.91	0.00	0.64	0.64
Model with PGx	12	54440.02	1.11	0.36	1.00
Model without the total number of medicines	11	54952.98	514.08	0.00	1.00
**Low Frailty**					
	K	AICc	Delta_AICc	AICc_Wt	Cum_Wt
Model without PGx	11	53189.98	0.00	0.71	0.71
Model with PGx	12	53191.81	1.83	0.29	1.00
Model without the total number of medicines	11	53334.38	144.40	0.00	1.00
**Repeat hospitalisation after a planned admission**					
**High Frailty**					
	K	AICc	Delta_AICc	AICc_Wt	Cum_Wt
Model with PGx	12	9978.40	0.00	0.95	0.95
Model without the total number of medicines	11	9984.95	6.55	0.04	0.99
Model without PGx	11	9987.07	8.67	0.01	1.00
**Intermediate Frailty**					
	K	AICc	Delta_AICc	AICc_Wt	Cum_Wt
Model with PGx	12	5243.22	0.00	0.55	0.55
Model without PGx	11	5243.60	0.38	0.45	1.00
Model without the total number of medicines	11	5266.30	23.08	0.00	1.00
**Low Frailty**					
	K	AICc	Delta_AICc	AICc_Wt	Cum_Wt
Model without the total number of medicines	11	10570.62	0.00	0.49	0.49
Model with PGx	12	10571.59	0.98	0.30	0.79
Model without PGx	11	10572.30	1.68	0.21	1.00

Delta_AICc = the difference in AIC score between the best model and the model being compared. A ‘Delta_AICc’ value greater than two indicates a clear significance, otherwise, two models are comparable.

K = is the number of independent variables.

Stratification by frailty status further showed that the best-fit model for unplanned admissions across all frailty groups incorporated the number of medications with PGx guidelines. For repeat hospital admissions following an unplanned admission, the best-fit model in high frailty patients included medications with PGx guidelines, with the lowest AICc score and carrying 100% of the cumulative model weight. In contrast, in intermediate and low frailty patients, the models excluding PGx demonstrated comparable predictive performance (Delta_AICc<2) to those incorporating PGx, carrying 36 and 29% of the cumulative model weight, respectively. Similar findings were noted for repeat hospital admission after a planned admission stratified by frailty status. The best-fit model in high frailty patients included medications with PGx guidelines, carrying 95% of the cumulative model weight. For intermediate and low frailty, the models without PGx were comparable in predictive performance (Delta_AICc<2) to those incorporating PGx, carrying 55 and 79% of the cumulative model weight, respectively.

## Discussion

Our study analysed 59,973 hospital admissions for 35,390 patients aged 65 and over admitted to hospital and show, as expected, extensive medicines use on admission, with 560,179 medicines appearing on the electronic health records. We found that 83% of these older patients were on polypharmacy, defined here as using five or more medicines. This prevalence is higher than the community setting where nearly half of individuals aged 65 and over are on polypharmacy [32], highlighting the increased likelihood of polypharmacy in hospitalised older people. Our data revealed that 69 medicines were prescribed amongst this population that have known PGx association and that 84% of patients admitted to hospital have one or more medications with PGx guidelines recorded in their electronic health record. This is similar to the findings in primary care where 80% of older people were exposed to at least one medication with PGx guidelines [25].

Patients with unplanned admission were more likely to be on medications with PGx guidelines compared to patients with planned admission, raising the question that the medicines may contribute to risk of hospitalisation. Indeed, published reports indicate that one in five hospital admissions in older patients occur due to their medicine use [2, 33, 34]. However, our dataset derived from coding in secondary care upon admission suggests that only 0.03% (Table 2) of admissions are known to be a result of medicines. Therefore, in this study, unplanned admissions are interpreted as a proxy for potential ADR-related admissions, acknowledging that this may underestimate the true number of ADR events. This discrepancy between the real time coding data suggests that the coding does not capture all cases. We note that in the research studies identification of admissions due to ADR, included expert review and consensus, causality assessments and additional checks to ensure no ADR cases were missed, using a resource that is not available during admission, particularly unplanned hospital admission. Consequently, due to these coding limitations, our study was unable to provide a detailed analysis specifically focused on ADR-related admissions. Future research using more comprehensive methods to identify ADR-related hospitalisations is warranted to better understand the role of ADRs and the potential impact of PGx-guided prescribing on reducing such admissions.

In our study we focussed on the concept that use of medications with PGx guidelines contribute to unexpected hospital admission and other adverse outcomes such as hospital length of stay and instances of repeat hospitalisation. Our models suggest that, in patients aged 65 and over who were prescribed at least one medicine upon admission, the number of medications with PGx guidelines was associated with an increased likelihood of adverse outcomes, alongside known risk factors such as frailty and total medication burden. In the multivariable prediction models, which consider the simultaneous effects of all predictors, frailty assessed using the HFRS emerged as the variable with the largest exponentiated coefficient, indicating it was the strongest contributor to adverse outcomes, including unplanned admission, prolonged hospital stay, and repeat hospitalisation. When further stratified by frailty status, our analysis indicated that the use of medications with PGx guidelines was associated with unplanned hospital admission across all patients. Additionally, in patients with high frailty status, use of medications with PGx guidelines was linked to longer hospital stays and repeat hospitalisations. These findings are important as they highlight potential risks associated with the use of medications with PGx guidelines in older patients. Our study aligns with Ashcraft et al. [35], which showed that unmanaged PGx risk relates to hospital length of stay.

The association shown here between the number of medications with PGx guidelines and adverse outcomes in high frailty patients suggests that this group is most susceptible to the risks associated with medications with PGx guidelines. While our data analysis cannot establish a direct causal link between the use of medications with PGx guidelines and adverse outcomes in patients living with frailty, they do emphasise the importance of considering frailty status when evaluating the impact of the use of medications with PGx guidelines on patient outcomes. When evaluating the link between frailty, polypharmacy and the use of medications with PGx guidelines, it is essential to recognise that individuals living with frailty are often exposed to a higher number of medicines due to their increased vulnerability to comorbidities and the need for complex treatment regimens [36]. Our data suggests that older patients living with frailty should be a priority group for implementation of PGx-guided prescribing.

This study highlights the quantitative importance of the use of medications with PGx guidelines in older people, with large numbers of individuals on multiple medications with PGx guidelines where genetic testing would lead to optimisation. Our data emphasises the need for rapid development of PGx-guided prescribing for older people, especially those living with frailty to enable personalised treatment plans. Assessing patients for PGx risk prior to hospitalisation and implementing PGx-guided medicine optimisation may help reduce hospital length of stay, lower healthcare costs, and improve patient quality of life. However, further research is needed to confirm these potential benefits.

The primary strength of our study is its contribution to understanding the relevance of medications with PGx guidelines in older patients and emphasising the potential need for their integration into routine clinical practice. Our findings suggest an association between the use of medications with PGx guidelines and adverse events such as unplanned hospital admissions and prolonged stays, which may increase the risk of complications like falls and pressure ulcers [37–40]. However, it is important to acknowledge the limitations of our study. First, our study did not include genomic information, preventing us from determining the exact proportion of patients who would benefit from PGx-guided care. Nonetheless, previous research indicates that a substantial proportion of the population (up to 95%) carries gene variants that influence drug response [19], and between 9 and 24% of individuals are prescribed medications that could be optimised through genetic testing [23, 41–43]. Additionally, our analysis did not account for primary and secondary diagnoses at admission or the total number of comorbidities, which are important factors that may influence the observed associations. An additional consideration is the role of drug-drug interactions (DDIs), particularly involving cytochrome P450 enzyme inhibitors and inducers, which can significantly influence drug metabolism and adverse event risk. While our study focused on drug-gene interactions, DDIs likely contribute alongside genetic factors to unplanned hospital admissions and other adverse outcomes. Due to limitations in the available data, we were unable to analyse DDIs in this study.

Another important consideration is medication changes following hospital discharge. Our dataset did not capture information on whether medications with PGx guidelines were discontinued, adjusted, or continued at discharge, nor their use at subsequent readmissions. Tracking these medication changes would be valuable to better understand the clinical management of medications with PGx guidelines and their impact on patient outcomes. Future studies incorporating longitudinal medication data across hospital episodes are warranted to explore this further.

Furthermore, while our study highlights an association between the use of medications with PGx guidelines and adverse outcomes, it does not establish causality, and because of the retrospective nature of our dataset, we were unable to control for all possible factors that might influence this association. Other unmeasured variables, such as underlying health conditions or the severity of illness, may have contributed to the observed outcomes. Finally, the dataset may underestimate the “sequence of admissions” for patients with index admissions later in the 24-month time period, potentially leading to conservative estimates of the association between exposure to medications with PGx guidelines and adverse outcomes. Despite these limitations, our study serves as an important step in evaluating the role of medications with PGx guidelines in older patients. Our findings highlight the need for further research to improve the use of PGx-guided treatments and evaluate their effects on patient health, particularly in frail older adults who are more vulnerable to medication-related complications.

Additionally, although we used measures such as residual sum of squares and AIC to assess model fit, we did not evaluate the absolute predictive performance of our models on independent data. Future research should include validation approaches such as cross-validation to estimate how well these models perform on new datasets, thereby confirming their generalisability and clinical utility. This step is essential to ensure the robustness of predictive models before implementation in practice.

## Conclusion

This study highlights the association between the use of medication(s) with PGx guidelines and adverse outcomes, particularly among highly frail patients. Hospital admission provides an opportunity to optimise medicines using PGx information, which may help reduce the risk of outcomes. Further research is needed to validate these findings and explore the practical implementation of PGx testing on a larger scale.

## Supplementary information

Supplementary Material

## Data Availability

The data that supports the findings of this study are available in the supplementary material of this article.

## Code availability

The analysis code cannot be shared publicly as it contains scripts tailored to NHS Trust confidential data, which are not shareable outside the secure research environment. Interested researchers may contact the corresponding author for more details about the analytical approach.
