# Supplementary material for: Use of medications with pharmacogenomic guidelines and adverse outcomes in hospitalised older patients: a retrospective cross-sectional study
Source: Pharmacogenomics J. 2026 Jan 28;26(1):3. doi: 10.1038/s41397-026-00396-3 (PMC12846911; doi:10.1038/s41397-026-00396-3)
Supplement: Supplementary file 1 — Supplementary Material [file 41397_2026_396_MOESM1_ESM.docx]

The appendix tables summarise key analyses: Table A1 details medication frequencies on admission, Table A2 tests for collinearity among predictors, Table A3 identifies the optimal number of predictor variables, Table A4 presents multivariable prediction equations for hospital outcomes, and Table A5 provides stratified prediction models based on frailty status, assessing length of stay and readmission risks.

**Appendix Table A1 The frequency of all the medicines recorded on patient admission to hospital.**

| **Drug Name** | **Number of appearances in dataset** | **Frequency** | **PGx/Non PGx** | **High risk/Not high risk** |
| --- | --- | --- | --- | --- |
| paracetamol | 27816 | 0.050600 | Non PGx | Not High Risk |
| lansoprazole | 21583 | 0.038530 | PGx | Not High Risk |
| atorvastatin | 19751 | 0.036000 | PGx | Not High Risk |
| bisoprolol | 17905 | 0.032600 | Non PGx | Not High Risk |
| salbutamol | 17032 | 0.031000 | Non PGx | Not High Risk |
| calcium+colecalciferol products | 16146 | 0.029400 | Non PGx | Not High Risk |
| furosemide | 13173 | 0.024000 | Non PGx | High Risk |
| ramipril | 12003 | 0.021900 | Non PGx | High Risk |
| simvastatin | 10803 | 0.019285 | PGx | Not High Risk |
| codeine | 10369 | 0.018511 | PGx | High Risk |
| folic acid | 10085 | 0.018400 | Non PGx | Not High Risk |
| omeprazole | 10009 | 0.017868 | PGx | Not High Risk |
| amlodipine | 9536 | 0.017400 | Non PGx | Not High Risk |
| insulin | 9237 | 0.016800 | Non PGx | Not High Risk |
| glyceryl | 8689 | 0.015800 | Non PGx | Not High Risk |
| levothyroxine | 8667 | 0.015800 | Non PGx | Not High Risk |
| metformin | 8232 | 0.015000 | Non PGx | Not High Risk |
| clopidogrel | 7767 | 0.013866 | PGx | High Risk |
| sennoside | 7283 | 0.013300 | Non PGx | Not High Risk |
| morphine | 5766 | 0.010500 | Non PGx | High Risk |
| warfarin | 5746 | 0.010258 | PGx | High Risk |
| Ferrous fumarate/gluconate/sulfate | 4949 | 0.009000 | Non PGx | Not High Risk |
| ranitidine | 4833 | 0.008800 | Non PGx | Not High Risk |
| amitriptyline | 4698 | 0.008387 | PGx | High Risk |
| carbocisteine | 4581 | 0.008300 | Non PGx | Not High Risk |
| allopurinol | 4462 | 0.007966 | PGx | Not High Risk |
| prednisolone | 4363 | 0.007900 | Non PGx | High Risk |
| ibuprofen | 4192 | 0.007484 | PGx | High Risk |
| Isosorbide dinitrate/mononitrate | 4055 | 0.007400 | Non PGx | Not High Risk |
| tamsulosin | 3952 | 0.007200 | Non PGx | Not High Risk |
| colecalciferol | 3774 | 0.006900 | Non PGx | Not High Risk |
| hydroxocobalamin | 3773 | 0.006900 | Non PGx | Not High Risk |
| losartan | 3755 | 0.006800 | Non PGx | High Risk |
| alendronate | 3694 | 0.006700 | Non PGx | Not High Risk |
| bumetanide | 3523 | 0.006400 | Non PGx | High Risk |
| gliclazide | 3518 | 0.006400 | Non PGx | Not High Risk |
| buprenorphine | 3384 | 0.006200 | Non PGx | High Risk |
| gabapentin | 3377 | 0.006100 | Non PGx | Not High Risk |
| sertraline | 3342 | 0.005966 | PGx | Not High Risk |
| lactulose | 3219 | 0.005900 | Non PGx | Not High Risk |
| docusate | 3186 | 0.005800 | Non PGx | Not High Risk |
| mirtazapine | 3065 | 0.005600 | Non PGx | High Risk |
| relvar ellipta | 3028 | 0.005500 | Non PGx | Not High Risk |
| pregabalin | 2992 | 0.005400 | Non PGx | Not High Risk |
| doxazosin | 2917 | 0.005300 | Non PGx | Not High Risk |
| finasteride | 2874 | 0.005200 | Non PGx | Not High Risk |
| digoxin | 2752 | 0.005000 | Non PGx | High Risk |
| bendroflumethiazide | 2694 | 0.004900 | Non PGx | High Risk |
| citalopram | 2663 | 0.004754 | PGx | High Risk |
| spironolactone | 2587 | 0.004700 | Non PGx | High Risk |
| candesartan | 2548 | 0.004600 | Non PGx | Not High Risk |
| quinine | 2386 | 0.004259 | PGx | Not High Risk |
| incruse ellipta | 2363 | 0.004300 | Non PGx | Not High Risk |
| atenolol | 2327 | 0.004200 | Non PGx | High Risk |
| tramadol | 2294 | 0.004095 | PGx | High Risk |
| loperamide | 2276 | 0.004100 | Non PGx | Not High Risk |
| rivaroxaban | 2262 | 0.004100 | Non PGx | High Risk |
| latanoprost | 2194 | 0.004000 | Non PGx | Not High Risk |
| salmeterol xinafoate preparations | 2144 | 0.003900 | Non PGx | Not High Risk |
| memantine | 2121 | 0.003900 | Non PGx | Not High Risk |
| anoro ellipta | 2075 | 0.003800 | Non PGx | Not High Risk |
| fostair | 2053 | 0.003700 | Non PGx | Not High Risk |
| diltiazem hydrochloride | 2018 | 0.003700 | Non PGx | Not High Risk |
| spiriva | 2004 | 0.003600 | Non PGx | Not High Risk |
| cetirizine | 1972 | 0.003600 | Non PGx | Not High Risk |
| donepezil | 1902 | 0.003500 | Non PGx | Not High Risk |
| thiamine | 1877 | 0.003400 | Non PGx | Not High Risk |
| solifenacin | 1866 | 0.003400 | Non PGx | Not High Risk |
| amoxicillin | 1828 | 0.003300 | Non PGx | Not High Risk |
| lisinopril | 1797 | 0.003300 | Non PGx | Not High Risk |
| easyhaler | 1652 | 0.003000 | Non PGx | Not High Risk |
| dihydrocodeine | 1560 | 0.002800 | Non PGx | High Risk |
| metoclopramide | 1529 | 0.002800 | Non PGx | Not High Risk |
| zopiclone | 1523 | 0.002800 | Non PGx | Not High Risk |
| creon | 1506 | 0.002700 | Non PGx | Non PGx |
| indapamide | 1397 | 0.002500 | Non PGx | High Risk |
| diclofenac | 1383 | 0.002500 | Non PGx | High Risk |
| levetiracetam | 1367 | 0.002500 | Non PGx | Not High Risk |
| lercanidipine | 1356 | 0.002500 | Non PGx | Not High Risk |
| tinzaparin | 1353 | 0.002500 | Non PGx | Not High Risk |
| dexamethasone | 1341 | 0.002400 | Non PGx | Not High Risk |
| trelegy | 1325 | 0.002400 | Non PGx | Not High Risk |
| bimatoprost | 1317 | 0.002400 | Non PGx | Not High Risk |
| hydrocortisone | 1316 | 0.002400 | Non PGx | Not High Risk |
| rosuvastatin | 1306 | 0.002331 | PGx | Not High Risk |
| sitagliptin | 1266 | 0.002300 | Non PGx | Not High Risk |
| linagliptin | 1244 | 0.002300 | Non PGx | Not High Risk |
| nicorandil | 1213 | 0.002200 | Non PGx | Not High Risk |
| fluoxetine | 1210 | 0.002200 | Non PGx | High Risk |
| perindopril | 1195 | 0.002200 | Non PGx | Not High Risk |
| nitrofurantoin | 1127 | 0.002012 | PGx | Not High Risk |
| methotrexate | 1102 | 0.002000 | Non PGx | Not High Risk |
| cyclizine | 1096 | 0.002000 | Non PGx | Not High Risk |
| hyoscine | 1082 | 0.002000 | Non PGx | Not High Risk |
| diazepam | 1066 | 0.001900 | Non PGx | Not High Risk |
| symbicort | 1047 | 0.001900 | Non PGx | Not High Risk |
| ezetimibe | 1044 | 0.001900 | Non PGx | Not High Risk |
| ondansetron | 1040 | 0.001857 | PGx | Not High Risk |
| carbidopa | 1020 | 0.001900 | Non PGx | Not High Risk |
| lorazepam | 1019 | 0.001900 | Non PGx | Not High Risk |
| mometasone | 1018 | 0.001900 | Non PGx | Not High Risk |
| felodipine | 1014 | 0.001800 | Non PGx | Not High Risk |
| montelukast | 1011 | 0.001800 | Non PGx | Not High Risk |
| ticagrelor | 1007 | 0.001800 | Non PGx | Not High Risk |
| beclomethasone diproprionate | 1002 | 0.001800 | Non PGx | Not High Risk |
| clenil modulite | 981 | 0.001800 | Non PGx | Not High Risk |
| sulfamethoxazole/trimethoprim | 967 | 0.001726 | PGx | Not High Risk |
| naproxen | 938 | 0.001700 | Non PGx | High Risk |
| betamethasone | 929 | 0.001700 | Non PGx | Not High Risk |
| fluticasone | 925 | 0.001700 | Non PGx | Not High Risk |
| fludrocortisone | 909 | 0.001700 | Non PGx | Not High Risk |
| fentanyl | 887 | 0.001600 | Non PGx | High Risk |
| doxycycline | 884 | 0.001600 | Non PGx | Not High Risk |
| pantoprazole | 871 | 0.001555 | PGx | Not High Risk |
| venlafaxine | 870 | 0.001553 | PGx | High Risk |
| pravastatin | 869 | 0.001600 | Non PGx | Not High Risk |
| piroxicam | 857 | 0.001530 | PGx | Not High Risk |
| benserazide | 844 | 0.001500 | Non PGx | Not High Risk |
| irbesartan | 817 | 0.001500 | Non PGx | High Risk |
| carbamazepine | 816 | 0.001457 | PGx | Not High Risk |
| fexofenadine | 810 | 0.001500 | Non PGx | Not High Risk |
| mebeverine | 772 | 0.001400 | Non PGx | Not High Risk |
| edoxaban | 770 | 0.001400 | Non PGx | Not High Risk |
| vitamins | 761 | 0.001400 | Non PGx | Not High Risk |
| nifedipine | 750 | 0.001400 | Non PGx | Not High Risk |
| betahistine | 725 | 0.001300 | Non PGx | Not High Risk |
| esomeprazole | 724 | 0.001300 | Non PGx | Not High Risk |
| brinzolamide | 723 | 0.001300 | Non PGx | Not High Risk |
| aciclovir | 717 | 0.001300 | Non PGx | Not High Risk |
| propranolol | 716 | 0.001300 | Non PGx | Not High Risk |
| risperidone | 707 | 0.001262 | PGx | Not High Risk |
| eplerenone | 707 | 0.001300 | Non PGx | Not High Risk |
| loratadine | 685 | 0.001200 | Non PGx | Not High Risk |
| forceval | 680 | 0.001200 | Non PGx | Not High Risk |
| darbepoetin | 667 | 0.001200 | Non PGx | Not High Risk |
| terbutaline | 661 | 0.001200 | Non PGx | Not High Risk |
| lamotrigine | 659 | 0.001200 | Non PGx | Not High Risk |
| enalapril | 655 | 0.001200 | Non PGx | Not High Risk |
| amiloride | 653 | 0.001200 | Non PGx | Not High Risk |
| bisacodyl | 628 | 0.001100 | Non PGx | Not High Risk |
| flucloxacillin | 627 | 0.001100 | Non PGx | Not High Risk |
| amiodarone | 598 | 0.001100 | Non PGx | Not High Risk |
| oxybutynin | 580 | 0.001100 | Non PGx | Not High Risk |
| hydroxychloroquine | 576 | 0.001000 | Non PGx | Not High Risk |
| prochlorperazine | 568 | 0.001000 | Non PGx | Not High Risk |
| mesalazine | 567 | 0.001012 | PGx | Not High Risk |
| chlorphenamine | 565 | 0.001000 | Non PGx | Not High Risk |
| mirabegron | 563 | 0.001000 | Non PGx | Not High Risk |
| trimbow | 553 | 0.001000 | Non PGx | Not High Risk |
| alfuzosin hydrochloride | 544 | 0.001000 | Non PGx | Not High Risk |
| azithromycin | 534 | 0.001000 | Non PGx | Not High Risk |
| temazepam | 522 | 0.001000 | Non PGx | Not High Risk |
| tolterodine | 516 | 0.000900 | Non PGx | Not High Risk |
| duloxetine | 514 | 0.000900 | Non PGx | Not High Risk |
| glimepiride | 503 | 0.000898 | PGx | Not High Risk |
| clarithromycin | 495 | 0.000900 | Non PGx | Not High Risk |
| sildenafil | 492 | 0.000900 | Non PGx | Not High Risk |
| olanzapine | 484 | 0.000900 | Non PGx | High Risk |
| baclofen | 466 | 0.000800 | Non PGx | Not High Risk |
| nebivolol | 458 | 0.000800 | Non PGx | Not High Risk |
| rivastigmine | 454 | 0.000800 | Non PGx | Not High Risk |
| valproate | 452 | 0.000807 | PGx | Not High Risk |
| valsartan | 450 | 0.000800 | Non PGx | Not High Risk |
| renavit | 444 | 0.000800 | Non PGx | Not High Risk |
| dorzolamide | 435 | 0.000800 | Non PGx | Not High Risk |
| sodium valproate | 431 | 0.000800 | Non PGx | Not High Risk |
| clotrimazole | 427 | 0.000800 | Non PGx | Not High Risk |
| uniphyllin | 427 | 0.000800 | Non PGx | Not High Risk |
| ranolazine | 424 | 0.000800 | Non PGx | Not High Risk |
| enoxaparin | 423 | 0.000800 | Non PGx | High Risk |
| ropinirole | 415 | 0.000800 | Non PGx | Not High Risk |
| trospium | 409 | 0.000700 | Non PGx | Not High Risk |
| trazodone | 407 | 0.000700 | Non PGx | Not High Risk |
| phenytoin | 404 | 0.000721 | PGx | Not High Risk |
| paroxetine | 398 | 0.000711 | PGx | High Risk |
| carvedilol | 398 | 0.000700 | Non PGx | Not High Risk |
| pivmecillinam | 396 | 0.000700 | Non PGx | Not High Risk |
| prostap | 390 | 0.000700 | Non PGx | Not High Risk |
| metoprolol | 385 | 0.000700 | Non PGx | Not High Risk |
| anastrozole | 367 | 0.000700 | Non PGx | Not High Risk |
| avamys | 357 | 0.000600 | Non PGx | Not High Risk |
| ivabradine | 356 | 0.000600 | Non PGx | Not High Risk |
| cyanocobalamin | 345 | 0.000600 | Non PGx | Not High Risk |
| bicalutamide | 336 | 0.000600 | Non PGx | Not High Risk |
| sulfasalazine | 335 | 0.000598 | PGx | Not High Risk |
| hydroxyzine | 334 | 0.000600 | Non PGx | Not High Risk |
| levomepromazine | 329 | 0.000600 | Non PGx | Not High Risk |
| nystatin | 325 | 0.000600 | Non PGx | Not High Risk |
| potassium | 325 | 0.000600 | Non PGx | Not High Risk |
| ursodeoxycholic | 325 | 0.000600 | Non PGx | Not High Risk |
| tacrolimus | 324 | 0.000578 | PGx | Not High Risk |
| ipratropium | 314 | 0.000600 | Non PGx | Not High Risk |
| clobetasol | 313 | 0.000600 | Non PGx | Not High Risk |
| risedronate | 311 | 0.000600 | Non PGx | Not High Risk |
| ciprofloxacin | 309 | 0.000552 | PGx | Not High Risk |
| hydroxycarbamide | 308 | 0.000600 | Non PGx | Not High Risk |
| aclidinium bromide preparations | 306 | 0.000600 | Non PGx | Not High Risk |
| quetiapine | 304 | 0.000600 | Non PGx | Not High Risk |
| azathioprine | 294 | 0.000525 | PGx | Not High Risk |
| leuprorelin | 290 | 0.000500 | Non PGx | Not High Risk |
| nitrazepam | 275 | 0.000500 | Non PGx | Not High Risk |
| naftidrofuryl | 273 | 0.000500 | Non PGx | Not High Risk |
| tiotropium | 269 | 0.000500 | Non PGx | Not High Risk |
| mycophenolate | 268 | 0.000478 | PGx | Not High Risk |
| clonazepam | 268 | 0.000500 | Non PGx | Not High Risk |
| balneum | 265 | 0.000500 | Non PGx | Not High Risk |
| procyclidine | 257 | 0.000500 | Non PGx | Not High Risk |
| benzydamine | 247 | 0.000400 | Non PGx | Not High Risk |
| lithium | 246 | 0.000400 | Non PGx | Not High Risk |
| magnesium | 245 | 0.000400 | Non PGx | Not High Risk |
| letrozole | 244 | 0.000400 | Non PGx | Not High Risk |
| movelat | 244 | 0.000400 | Non PGx | Not High Risk |
| iron isomaltoside/sucrose | 241 | 0.000400 | Non PGx | Not High Risk |
| priadel | 233 | 0.000400 | Non PGx | Not High Risk |
| timolol | 232 | 0.000400 | Non PGx | Not High Risk |
| dioralyte | 230 | 0.000400 | Non PGx | Not High Risk |
| verapamil | 222 | 0.000400 | Non PGx | Not High Risk |
| denosumab | 218 | 0.000400 | Non PGx | Not High Risk |
| promethazine | 217 | 0.000400 | Non PGx | Not High Risk |
| chloramphenicol | 216 | 0.000386 | PGx | Not High Risk |
| pramipexole | 215 | 0.000400 | Non PGx | Not High Risk |
| colchicine | 210 | 0.000400 | Non PGx | Not High Risk |
| midazolam | 208 | 0.000400 | Non PGx | Not High Risk |
| phenoxymethylpenicillin | 207 | 0.000400 | Non PGx | Not High Risk |
| ketoconazole | 206 | 0.000400 | Non PGx | Not High Risk |
| lidocaine | 206 | 0.000400 | Non PGx | Not High Risk |
| pioglitazone | 199 | 0.000400 | Non PGx | Not High Risk |
| brimonidine | 197 | 0.000400 | Non PGx | Not High Risk |
| liraglutide | 196 | 0.000400 | Non PGx | Not High Risk |
| mupirocin | 194 | 0.000400 | Non PGx | Not High Risk |
| dipyridamole | 193 | 0.000400 | Non PGx | Not High Risk |
| cinacalcet | 191 | 0.000300 | Non PGx | Not High Risk |
| moxonidine | 190 | 0.000300 | Non PGx | Not High Risk |
| sevelamer | 187 | 0.000300 | Non PGx | Not High Risk |
| stalevo | 187 | 0.000300 | Non PGx | Not High Risk |
| carbimazole | 186 | 0.000300 | Non PGx | Not High Risk |
| dosulepin | 186 | 0.000300 | Non PGx | Not High Risk |
| zoledronic | 186 | 0.000300 | Non PGx | Not High Risk |
| budesonide | 181 | 0.000300 | Non PGx | Not High Risk |
| dapagliflozin | 179 | 0.000300 | Non PGx | Not High Risk |
| lacidipine | 177 | 0.000300 | Non PGx | Not High Risk |
| estradiol | 176 | 0.000300 | Non PGx | Not High Risk |
| fluconazole | 175 | 0.000300 | Non PGx | Not High Risk |
| sodium cromoglicate | 174 | 0.000300 | Non PGx | Not High Risk |
| galantamine | 173 | 0.000300 | Non PGx | Not High Risk |
| tadalafil | 173 | 0.000300 | Non PGx | Not High Risk |
| rabeprazole | 168 | 0.000300 | Non PGx | Not High Risk |
| primidone | 166 | 0.000300 | Non PGx | Not High Risk |
| cefalexin | 165 | 0.000300 | Non PGx | Not High Risk |
| clobetasone | 165 | 0.000300 | Non PGx | Not High Risk |
| nortriptyline | 164 | 0.000293 | PGx | High Risk |
| clinitas | 161 | 0.000300 | Non PGx | Not High Risk |
| zolpidem | 161 | 0.000300 | Non PGx | Not High Risk |
| glandosane | 160 | 0.000300 | Non PGx | Not High Risk |
| haloperidol | 159 | 0.000300 | Non PGx | Not High Risk |
| celecoxib | 151 | 0.000270 | PGx | High Risk |
| voltarol | 149 | 0.000300 | Non PGx | Not High Risk |
| escitalopram | 147 | 0.000262 | PGx | High Risk |
| aripiprazole | 146 | 0.000261 | PGx | Not High Risk |
| timodine | 146 | 0.000300 | Non PGx | Not High Risk |
| metronidazole | 145 | 0.000300 | Non PGx | Not High Risk |
| meloxicam | 141 | 0.000252 | PGx | Not High Risk |
| flupentixol | 141 | 0.000300 | Non PGx | Not High Risk |
| domperidone | 139 | 0.000300 | Non PGx | Not High Risk |
| ganfort | 139 | 0.000300 | Non PGx | Not High Risk |
| tranexamic | 139 | 0.000300 | Non PGx | Not High Risk |
| cinnarizine | 138 | 0.000300 | Non PGx | Not High Risk |
| monomil | 137 | 0.000200 | Non PGx | Not High Risk |
| empagliflozin | 135 | 0.000200 | Non PGx | Not High Risk |
| amisulpride | 132 | 0.000200 | Non PGx | Not High Risk |
| fusidic | 130 | 0.000200 | Non PGx | Not High Risk |
| neoral | 130 | 0.000200 | Non PGx | Not High Risk |
| spiolto | 130 | 0.000200 | Non PGx | Not High Risk |
| hydralazine | 128 | 0.000229 | PGx | Not High Risk |
| epoetin | 128 | 0.000200 | Non PGx | Not High Risk |
| rasagiline | 126 | 0.000200 | Non PGx | Not High Risk |
| dabigatran | 125 | 0.000200 | Non PGx | Not High Risk |
| levofloxacin | 123 | 0.000200 | Non PGx | Not High Risk |
| sinemet | 123 | 0.000200 | Non PGx | Not High Risk |
| rifaximin | 122 | 0.000200 | Non PGx | Not High Risk |
| seebri | 122 | 0.000200 | Non PGx | Not High Risk |
| estriol | 120 | 0.000200 | Non PGx | Not High Risk |
| tamoxifen | 119 | 0.000212 | PGx | Not High Risk |
| itraconazole | 118 | 0.000200 | Non PGx | Not High Risk |
| flecainide | 117 | 0.000200 | Non PGx | Not High Risk |
| hydrochlorothiazide | 117 | 0.000200 | Non PGx | Not High Risk |
| bezafibrate | 116 | 0.000200 | Non PGx | Not High Risk |
| colestyramine | 115 | 0.000200 | Non PGx | Not High Risk |
| micralax | 115 | 0.000200 | Non PGx | Not High Risk |
| terbinafine | 114 | 0.000200 | Non PGx | Not High Risk |
| toujeo | 114 | 0.000200 | Non PGx | Not High Risk |
| methenamine | 111 | 0.000200 | Non PGx | Not High Risk |
| clinical | 109 | 0.000200 | Non PGx | Not High Risk |
| etoricoxib | 109 | 0.000200 | Non PGx | Not High Risk |
| betnovate | 108 | 0.000200 | Non PGx | Not High Risk |
| braltus | 108 | 0.000200 | Non PGx | Not High Risk |
| testosterone | 108 | 0.000200 | Non PGx | Not High Risk |
| sacubitril | 106 | 0.000200 | Non PGx | Not High Risk |
| dextran | 105 | 0.000200 | Non PGx | Not High Risk |
| fluvastatin | 104 | 0.000200 | Non PGx | Not High Risk |
| madopar | 103 | 0.000200 | Non PGx | Not High Risk |
| miconazole | 102 | 0.000200 | Non PGx | Not High Risk |
| phenobarbital | 102 | 0.000200 | Non PGx | Not High Risk |
| telmisartan | 102 | 0.000200 | Non PGx | Not High Risk |
| zuclopenthixol | 102 | 0.000200 | Non PGx | Not High Risk |
| metolazone | 100 | 0.000200 | Non PGx | Not High Risk |
| zoladex | 100 | 0.000200 | Non PGx | Not High Risk |
| co-dydramol | 96 | 0.000200 | Non PGx | Not High Risk |
| olmesartan | 93 | 0.000200 | Non PGx | Not High Risk |
| alogliptin | 92 | 0.000200 | Non PGx | Not High Risk |
| canagliflozin | 92 | 0.000200 | Non PGx | Not High Risk |
| pizotifen | 91 | 0.000200 | Non PGx | Not High Risk |
| lofepramine | 89 | 0.000200 | Non PGx | Not High Risk |
| ketoprofen | 88 | 0.000200 | Non PGx | Not High Risk |
| mucopolysaccharide | 87 | 0.000200 | Non PGx | Not High Risk |
| chlorpromazine | 86 | 0.000200 | Non PGx | Not High Risk |
| flutiform | 86 | 0.000200 | Non PGx | Not High Risk |
| topiramate | 86 | 0.000200 | Non PGx | Not High Risk |
| butec | 85 | 0.000200 | Non PGx | Not High Risk |
| sotalol | 85 | 0.000200 | Non PGx | Not High Risk |
| benzalkonium | 84 | 0.000200 | Non PGx | Not High Risk |
| ketorolac trometamol | 84 | 0.000200 | Non PGx | Not High Risk |
| dermovate | 82 | 0.000100 | Non PGx | Not High Risk |
| sodium feredetate | 82 | 0.000100 | Non PGx | Not High Risk |
| sumatriptan | 82 | 0.000100 | Non PGx | Not High Risk |
| goserelin | 81 | 0.000100 | Non PGx | Not High Risk |
| dutasteride | 80 | 0.000100 | Non PGx | Not High Risk |
| aminophylline | 79 | 0.000100 | Non PGx | Not High Risk |
| pyridostigmine | 78 | 0.000100 | Non PGx | Not High Risk |
| desmopressin | 76 | 0.000100 | Non PGx | Not High Risk |
| saxagliptin | 76 | 0.000100 | Non PGx | Not High Risk |
| clomipramine | 75 | 0.000134 | PGx | Not High Risk |
| travoprost | 75 | 0.000100 | Non PGx | Not High Risk |
| bricanyl | 74 | 0.000100 | Non PGx | Not High Risk |
| dalteparin | 73 | 0.000100 | Non PGx | Not High Risk |
| diamorphine | 73 | 0.000100 | Non PGx | Not High Risk |
| flixotide | 73 | 0.000100 | Non PGx | Not High Risk |
| diethylamine | 72 | 0.000100 | Non PGx | Not High Risk |
| tresiba | 71 | 0.000100 | Non PGx | Not High Risk |
| cinchocaine | 70 | 0.000100 | Non PGx | Not High Risk |
| febuxostat | 70 | 0.000100 | Non PGx | Not High Risk |
| midodrine | 68 | 0.000100 | Non PGx | Not High Risk |
| pulmicort | 66 | 0.000100 | Non PGx | Not High Risk |
| nefopam | 65 | 0.000100 | Non PGx | Not High Risk |
| beconase | 64 | 0.000100 | Non PGx | Not High Risk |
| dovobet | 64 | 0.000100 | Non PGx | Not High Risk |
| eprex | 64 | 0.000100 | Non PGx | Not High Risk |
| filgrastim | 64 | 0.000100 | Non PGx | Not High Risk |
| leflunomide | 64 | 0.000100 | Non PGx | Not High Risk |
| calcipotriol | 63 | 0.000100 | Non PGx | Not High Risk |
| duoresp | 63 | 0.000100 | Non PGx | Not High Risk |
| tizanidine | 63 | 0.000100 | Non PGx | Not High Risk |
| ultrabase | 63 | 0.000100 | Non PGx | Not High Risk |
| fenofibrate | 61 | 0.000100 | Non PGx | Not High Risk |
| oseltamivir | 61 | 0.000100 | Non PGx | Not High Risk |
| cefradine | 59 | 0.000100 | Non PGx | Not High Risk |
| daktacort | 59 | 0.000100 | Non PGx | Not High Risk |
| hirudoid | 59 | 0.000100 | Non PGx | Not High Risk |
| vagifem | 59 | 0.000100 | Non PGx | Not High Risk |
| alverine | 58 | 0.000100 | Non PGx | Not High Risk |
| cleen | 58 | 0.000100 | Non PGx | Not High Risk |
| imatinib | 58 | 0.000100 | Non PGx | Not High Risk |
| lauromacrogols | 58 | 0.000100 | Non PGx | Not High Risk |
| slo-phyllin | 58 | 0.000100 | Non PGx | Not High Risk |
| amorolfine | 57 | 0.000100 | Non PGx | Not High Risk |
| crotamiton | 57 | 0.000100 | Non PGx | Not High Risk |
| etanercept | 57 | 0.000100 | Non PGx | Not High Risk |
| lenalidomide | 57 | 0.000100 | Non PGx | Not High Risk |
| monopost | 57 | 0.000100 | Non PGx | Not High Risk |
| sodium ascorbate | 56 | 0.000100 | Non PGx | Not High Risk |
| acetylcysteine | 55 | 0.000100 | Non PGx | Not High Risk |
| difflam | 55 | 0.000100 | Non PGx | Not High Risk |
| lymecycline | 55 | 0.000100 | Non PGx | Not High Risk |
| erythromycin | 54 | 0.000100 | Non PGx | Not High Risk |
| naseptin | 54 | 0.000100 | Non PGx | Not High Risk |
| clobazam | 53 | 0.000100 | Non PGx | Not High Risk |
| elantan | 53 | 0.000100 | Non PGx | Not High Risk |
| pyridoxine | 53 | 0.000100 | Non PGx | Not High Risk |
| zinc | 53 | 0.000100 | Non PGx | Not High Risk |
| cimetidine | 52 | 0.000100 | Non PGx | Not High Risk |
| clonidine | 52 | 0.000100 | Non PGx | Not High Risk |
| colomycin | 52 | 0.000100 | Non PGx | Not High Risk |
| cosopt | 52 | 0.000100 | Non PGx | Not High Risk |
| sando-k | 52 | 0.000100 | Non PGx | Not High Risk |
| atropine | 51 | 0.000100 | Non PGx | Not High Risk |
| phyllocontin | 51 | 0.000100 | Non PGx | Not High Risk |
| imipramine | 50 | 0.000089 | PGx | Not High Risk |
| exemestane | 50 | 0.000100 | Non PGx | Not High Risk |
| sukkarto | 50 | 0.000100 | Non PGx | Not High Risk |
| nintedanib | 49 | 0.000100 | Non PGx | Not High Risk |
| methyldopa | 48 | 0.000100 | Non PGx | Not High Risk |
| fluorouracil | 47 | 0.000084 | PGx | Not High Risk |
| adrenaline | 47 | 0.000100 | Non PGx | Not High Risk |
| alphosyl | 47 | 0.000100 | Non PGx | Not High Risk |
| indometacin | 47 | 0.000100 | Non PGx | Not High Risk |
| naloxone | 47 | 0.000100 | Non PGx | Not High Risk |
| rotigotine | 47 | 0.000100 | Non PGx | Not High Risk |
| dapsone | 46 | 0.000082 | PGx | Not High Risk |
| diprosalic | 46 | 0.000100 | Non PGx | Not High Risk |
| maxidex | 46 | 0.000100 | Non PGx | Not High Risk |
| melatonin | 46 | 0.000100 | Non PGx | Not High Risk |
| capecitabine | 45 | 0.000080 | PGx | Not High Risk |
| exenatide | 45 | 0.000100 | Non PGx | Not High Risk |
| methocarbamol | 45 | 0.000100 | Non PGx | Not High Risk |
| ruxolitinib | 45 | 0.000100 | Non PGx | Not High Risk |
| isotard | 44 | 0.000100 | Non PGx | Not High Risk |
| quinapril | 44 | 0.000100 | Non PGx | Not High Risk |
| theophylline | 44 | 0.000100 | Non PGx | Not High Risk |
| tetrabenazine | 43 | 0.000077 | PGx | Not High Risk |
| amantadine | 43 | 0.000100 | Non PGx | Not High Risk |
| artelac | 43 | 0.000100 | Non PGx | Not High Risk |
| cyclophosphamide | 43 | 0.000100 | Non PGx | Not High Risk |
| proctosedyl | 43 | 0.000100 | Non PGx | Not High Risk |
| urea | 43 | 0.000100 | Non PGx | Not High Risk |
| zarzio | 42 | 0.000100 | Non PGx | Not High Risk |
| colesevelam | 41 | 0.000100 | Non PGx | Not High Risk |
| fultium | 41 | 0.000100 | Non PGx | Not High Risk |
| keppra | 41 | 0.000100 | Non PGx | Not High Risk |
| senna | 41 | 0.000100 | Non PGx | Not High Risk |
| clindamycin | 40 | 0.000100 | Non PGx | Not High Risk |
| entresto | 40 | 0.000100 | Non PGx | Not High Risk |
| ibandronate | 40 | 0.000100 | Non PGx | Not High Risk |
| sucralfate | 40 | 0.000100 | Non PGx | Not High Risk |
| dulaglutide | 39 | 0.000100 | Non PGx | Not High Risk |
| emerade | 39 | 0.000100 | Non PGx | Not High Risk |
| scheriproct | 39 | 0.000100 | Non PGx | Not High Risk |
| ascorbic | 38 | 0.000100 | Non PGx | Not High Risk |
| depo-medrone | 38 | 0.000100 | Non PGx | Not High Risk |
| dermatonics | 38 | 0.000100 | Non PGx | Not High Risk |
| calmurid | 37 | 0.000100 | Non PGx | Not High Risk |
| diafer | 37 | 0.000100 | Non PGx | Not High Risk |
| oxazepam | 37 | 0.000100 | Non PGx | Not High Risk |
| eprosartan | 36 | 0.000100 | Non PGx | Not High Risk |
| mepilex | 36 | 0.000100 | Non PGx | Not High Risk |
| piperacillin | 36 | 0.000100 | Non PGx | Not High Risk |
| victoza | 36 | 0.000100 | Non PGx | Not High Risk |
| acitretin | 35 | 0.000100 | Non PGx | Not High Risk |
| ciclosporin | 35 | 0.000100 | Non PGx | Not High Risk |
| drapolene | 35 | 0.000100 | Non PGx | Not High Risk |
| pentoxifylline | 35 | 0.000100 | Non PGx | Not High Risk |
| riluzole | 35 | 0.000100 | Non PGx | Not High Risk |
| rizatriptan | 35 | 0.000100 | Non PGx | Not High Risk |
| aranesp | 34 | 0.000100 | Non PGx | Not High Risk |
| azopt | 34 | 0.000100 | Non PGx | Not High Risk |
| captopril | 34 | 0.000100 | Non PGx | Not High Risk |
| fucibet | 34 | 0.000100 | Non PGx | Not High Risk |
| doxepin | 33 | 0.000059 | PGx | Not High Risk |
| gentamicin | 33 | 0.000059 | PGx | Not High Risk |
| lanreotide | 33 | 0.000100 | Non PGx | Not High Risk |
| oxytetracycline | 33 | 0.000100 | Non PGx | Not High Risk |
| sodium clodronate | 33 | 0.000100 | Non PGx | Not High Risk |
| nicardipine | 32 | 0.000100 | Non PGx | Not High Risk |
| nutrison | 32 | 0.000100 | Non PGx | Not High Risk |
| umeclidinium | 32 | 0.000100 | Non PGx | Not High Risk |
| alvesco | 31 | 0.000100 | Non PGx | Not High Risk |
| prasugrel | 31 | 0.000100 | Non PGx | Not High Risk |
| ergocalciferol | 30 | 0.000100 | Non PGx | Not High Risk |
| valganciclovir | 30 | 0.000100 | Non PGx | Not High Risk |
| bonjela | 29 | 0.000100 | Non PGx | Not High Risk |
| calcitriol | 29 | 0.000100 | Non PGx | Not High Risk |
| centrum | 29 | 0.000100 | Non PGx | Not High Risk |
| darifenacin | 29 | 0.000100 | Non PGx | Not High Risk |
| docosahexaenoic | 29 | 0.000100 | Non PGx | Not High Risk |
| eumovate | 29 | 0.000100 | Non PGx | Not High Risk |
| eurax | 29 | 0.000100 | Non PGx | Not High Risk |
| posaconazole | 29 | 0.000100 | Non PGx | Not High Risk |
| azelastine | 28 | 0.000100 | Non PGx | Not High Risk |
| betnesol | 28 | 0.000100 | Non PGx | Not High Risk |
| clozapine | 28 | 0.000100 | Non PGx | Not High Risk |
| cyproterone | 28 | 0.000100 | Non PGx | Not High Risk |
| fesoterodine | 28 | 0.000100 | Non PGx | Not High Risk |
| lormetazepam | 28 | 0.000100 | Non PGx | Not High Risk |
| teicoplanin | 28 | 0.000100 | Non PGx | Not High Risk |
| triamcinolone | 28 | 0.000100 | Non PGx | Not High Risk |
| ultibro | 28 | 0.000100 | Non PGx | Not High Risk |
| varenicline | 28 | 0.000100 | Non PGx | Not High Risk |
| elocon | 27 | 0.000000 | Non PGx | Not High Risk |
| enzalutamide | 27 | 0.000000 | Non PGx | Not High Risk |
| pred forte eye drops | 27 | 0.000000 | Non PGx | Not High Risk |
| aflibercept | 26 | 0.000000 | Non PGx | Not High Risk |
| atrauman | 26 | 0.000000 | Non PGx | Not High Risk |
| canesten | 26 | 0.000000 | Non PGx | Not High Risk |
| cosmocol | 26 | 0.000000 | Non PGx | Not High Risk |
| evorel | 26 | 0.000000 | Non PGx | Not High Risk |
| fluorometholone | 26 | 0.000000 | Non PGx | Not High Risk |
| ibrutinib | 26 | 0.000000 | Non PGx | Not High Risk |
| nebido | 26 | 0.000000 | Non PGx | Not High Risk |
| trihexyphenidyl | 26 | 0.000000 | Non PGx | Not High Risk |
| acetazolamide | 25 | 0.000000 | Non PGx | Not High Risk |
| carmize | 25 | 0.000000 | Non PGx | Not High Risk |
| desloratadine | 25 | 0.000000 | Non PGx | Not High Risk |
| medroxyprogesterone | 25 | 0.000000 | Non PGx | Not High Risk |
| ofev | 25 | 0.000000 | Non PGx | Not High Risk |
| sanatogen | 25 | 0.000000 | Non PGx | Not High Risk |
| terazosin | 25 | 0.000000 | Non PGx | Not High Risk |
| torasemide | 25 | 0.000000 | Non PGx | Not High Risk |
| trimovate | 25 | 0.000000 | Non PGx | Not High Risk |
| vildagliptin | 25 | 0.000000 | Non PGx | Not High Risk |
| duraphat | 24 | 0.000000 | Non PGx | Not High Risk |
| fosfomycin | 24 | 0.000000 | Non PGx | Not High Risk |
| selegiline | 24 | 0.000000 | Non PGx | Not High Risk |
| tenofovir | 24 | 0.000000 | Non PGx | Not High Risk |
| teriparatide | 24 | 0.000000 | Non PGx | Not High Risk |
| acenocoumarol | 23 | 0.000041 | PGx | Not High Risk |
| mercaptopurine | 23 | 0.000041 | PGx | Not High Risk |
| azarga | 23 | 0.000000 | Non PGx | Not High Risk |
| desunin | 23 | 0.000000 | Non PGx | Not High Risk |
| dulcolax | 23 | 0.000000 | Non PGx | Not High Risk |
| formoterol | 23 | 0.000000 | Non PGx | Not High Risk |
| ketovite | 23 | 0.000000 | Non PGx | Not High Risk |
| metanium | 23 | 0.000000 | Non PGx | Not High Risk |
| nebusal | 23 | 0.000000 | Non PGx | Not High Risk |
| nizatidine | 23 | 0.000000 | Non PGx | Not High Risk |
| oxerutins | 23 | 0.000000 | Non PGx | Not High Risk |
| propantheline | 23 | 0.000000 | Non PGx | Not High Risk |
| trifluoperazine | 23 | 0.000000 | Non PGx | Not High Risk |
| glipizide | 22 | 0.000039 | PGx | Not High Risk |
| celiprolol | 22 | 0.000000 | Non PGx | Not High Risk |
| degarelix | 22 | 0.000000 | Non PGx | Not High Risk |
| forticreme | 22 | 0.000000 | Non PGx | Not High Risk |
| glucosamine | 22 | 0.000000 | Non PGx | Not High Risk |
| isoniazid | 22 | 0.000000 | Non PGx | Not High Risk |
| lanthanum | 22 | 0.000000 | Non PGx | Not High Risk |
| naratriptan | 22 | 0.000000 | Non PGx | Not High Risk |
| octreotide | 22 | 0.000000 | Non PGx | Not High Risk |
| prucalopride | 22 | 0.000000 | Non PGx | Not High Risk |
| raloxifene | 22 | 0.000000 | Non PGx | Not High Risk |
| trandolapril | 22 | 0.000000 | Non PGx | Not High Risk |
| trulicity | 22 | 0.000000 | Non PGx | Not High Risk |
| uniroid | 22 | 0.000000 | Non PGx | Not High Risk |
| colpermin | 21 | 0.000000 | Non PGx | Not High Risk |
| flamazine | 21 | 0.000000 | Non PGx | Not High Risk |
| flixonase | 21 | 0.000000 | Non PGx | Not High Risk |
| ibandronic | 21 | 0.000000 | Non PGx | Not High Risk |
| linezolid | 21 | 0.000000 | Non PGx | Not High Risk |
| lumigan | 21 | 0.000000 | Non PGx | Not High Risk |
| pancrease | 21 | 0.000000 | Non PGx | Not High Risk |
| simbrinza | 21 | 0.000000 | Non PGx | Not High Risk |
| trusopt | 21 | 0.000000 | Non PGx | Not High Risk |
| chemydur | 20 | 0.000000 | Non PGx | Not High Risk |
| enstilar | 20 | 0.000000 | Non PGx | Not High Risk |
| glucophage | 20 | 0.000000 | Non PGx | Not High Risk |
| glutafin | 20 | 0.000000 | Non PGx | Not High Risk |
| glycopyrronium | 20 | 0.000000 | Non PGx | Not High Risk |
| naloxegol | 20 | 0.000000 | Non PGx | Not High Risk |
| pholcodine | 20 | 0.000000 | Non PGx | Not High Risk |
| pilocarpine | 20 | 0.000000 | Non PGx | Not High Risk |
| sandocal | 20 | 0.000000 | Non PGx | Not High Risk |
| selenium | 20 | 0.000000 | Non PGx | Not High Risk |
| apremilast | 19 | 0.000000 | Non PGx | Not High Risk |
| atrovent | 19 | 0.000000 | Non PGx | Not High Risk |
| colistimethate | 19 | 0.000000 | Non PGx | Not High Risk |
| corsodyl | 19 | 0.000000 | Non PGx | Not High Risk |
| diethylstilbestrol | 19 | 0.000000 | Non PGx | Not High Risk |
| eucerin | 19 | 0.000000 | Non PGx | Not High Risk |
| fondaparinux | 19 | 0.000000 | Non PGx | Not High Risk |
| huxd3 | 19 | 0.000000 | Non PGx | Not High Risk |
| indoramin | 19 | 0.000000 | Non PGx | Not High Risk |
| infliximab | 19 | 0.000000 | Non PGx | Not High Risk |
| isomol | 19 | 0.000000 | Non PGx | Not High Risk |
| maalox | 19 | 0.000000 | Non PGx | Not High Risk |
| megestrol | 19 | 0.000000 | Non PGx | Not High Risk |
| methylprednisolone | 19 | 0.000000 | Non PGx | Not High Risk |
| omacor | 19 | 0.000000 | Non PGx | Not High Risk |
| omega | 19 | 0.000000 | Non PGx | Not High Risk |
| questran | 19 | 0.000000 | Non PGx | Not High Risk |
| requip | 19 | 0.000000 | Non PGx | Not High Risk |
| thalidomide | 19 | 0.000000 | Non PGx | Not High Risk |
| dantron | 18 | 0.000000 | Non PGx | Not High Risk |
| prazosin | 18 | 0.000000 | Non PGx | Not High Risk |
| simeticone | 18 | 0.000000 | Non PGx | Not High Risk |
| tapentadol | 18 | 0.000000 | Non PGx | Not High Risk |
| tibolone | 18 | 0.000000 | Non PGx | Not High Risk |
| tocilizumab | 18 | 0.000000 | Non PGx | Not High Risk |
| aloflute | 17 | 0.000000 | Non PGx | Not High Risk |
| conjugated | 17 | 0.000000 | Non PGx | Not High Risk |
| diphenhydramine | 17 | 0.000000 | Non PGx | Not High Risk |
| entecavir | 17 | 0.000000 | Non PGx | Not High Risk |
| fucidin | 17 | 0.000000 | Non PGx | Not High Risk |
| half securon | 17 | 0.000000 | Non PGx | Not High Risk |
| invita | 17 | 0.000000 | Non PGx | Not High Risk |
| loprazolam | 17 | 0.000000 | Non PGx | Not High Risk |
| maxitrol | 17 | 0.000000 | Non PGx | Not High Risk |
| oxis | 17 | 0.000000 | Non PGx | Not High Risk |
| phosphate-sandoz | 17 | 0.000000 | Non PGx | Not High Risk |
| pomalidomide | 17 | 0.000000 | Non PGx | Not High Risk |
| premarin | 17 | 0.000000 | Non PGx | Not High Risk |
| regurin | 17 | 0.000000 | Non PGx | Not High Risk |
| safinamide | 17 | 0.000000 | Non PGx | Not High Risk |
| sodium dihydrogen | 17 | 0.000000 | Non PGx | Not High Risk |
| solaraze | 17 | 0.000000 | Non PGx | Not High Risk |
| aliskiren | 16 | 0.000000 | Non PGx | Not High Risk |
| altraplen | 16 | 0.000000 | Non PGx | Not High Risk |
| anagrelide | 16 | 0.000000 | Non PGx | Not High Risk |
| bosentan | 16 | 0.000000 | Non PGx | Not High Risk |
| cilostazol | 16 | 0.000000 | Non PGx | Not High Risk |
| glucagon | 16 | 0.000000 | Non PGx | Not High Risk |
| nedocromil | 16 | 0.000000 | Non PGx | Not High Risk |
| raltegravir | 16 | 0.000000 | Non PGx | Not High Risk |
| sastravi | 16 | 0.000000 | Non PGx | Not High Risk |
| sebco | 16 | 0.000000 | Non PGx | Not High Risk |
| survimed | 16 | 0.000000 | Non PGx | Not High Risk |
| thealoz | 16 | 0.000000 | Non PGx | Not High Risk |
| abiraterone | 15 | 0.000000 | Non PGx | Not High Risk |
| ciprofibrate | 15 | 0.000000 | Non PGx | Not High Risk |
| dolutegravir | 15 | 0.000000 | Non PGx | Not High Risk |
| emla | 15 | 0.000000 | Non PGx | Not High Risk |
| etodolac | 15 | 0.000000 | Non PGx | Not High Risk |
| glucogel | 15 | 0.000000 | Non PGx | Not High Risk |
| inadine | 15 | 0.000000 | Non PGx | Not High Risk |
| lacosamide | 15 | 0.000000 | Non PGx | Not High Risk |
| manevac | 15 | 0.000000 | Non PGx | Not High Risk |
| neditol | 15 | 0.000000 | Non PGx | Not High Risk |
| ovestin | 15 | 0.000000 | Non PGx | Not High Risk |
| sirolimus | 15 | 0.000000 | Non PGx | Not High Risk |
| taurolock | 15 | 0.000000 | Non PGx | Not High Risk |
| vancomycin | 15 | 0.000000 | Non PGx | Not High Risk |
| voriconazole | 14 | 0.000000 | PGx | Not High Risk |
| acetic acid | 14 | 0.000000 | Non PGx | Not High Risk |
| ambisome | 14 | 0.000000 | Non PGx | Not High Risk |
| buscopan | 14 | 0.000000 | Non PGx | Not High Risk |
| dabrafenib | 14 | 0.000000 | Non PGx | Not High Risk |
| deximune | 14 | 0.000000 | Non PGx | Not High Risk |
| dovonex | 14 | 0.000000 | Non PGx | Not High Risk |
| emtricitabine | 14 | 0.000000 | Non PGx | Not High Risk |
| normacol | 14 | 0.000000 | Non PGx | Not High Risk |
| orlistat | 14 | 0.000000 | Non PGx | Not High Risk |
| renacet | 14 | 0.000000 | Non PGx | Not High Risk |
| shortec | 14 | 0.000000 | Non PGx | Not High Risk |
| valaciclovir | 14 | 0.000000 | Non PGx | Not High Risk |
| vardenafil | 14 | 0.000000 | Non PGx | Not High Risk |
| chlordiazepoxide | 13 | 0.000000 | Non PGx | Not High Risk |
| dantrolene | 13 | 0.000000 | Non PGx | Not High Risk |
| dried aluminium | 13 | 0.000000 | Non PGx | Not High Risk |
| evacal | 13 | 0.000000 | Non PGx | Not High Risk |
| fluocinolone | 13 | 0.000000 | Non PGx | Not High Risk |
| heparin | 13 | 0.000000 | Non PGx | Not High Risk |
| kenalog | 13 | 0.000000 | Non PGx | Not High Risk |
| lactic + salicylcic acid topical product | 13 | 0.000000 | Non PGx | Not High Risk |
| nabumetone | 13 | 0.000000 | Non PGx | Not High Risk |
| omalizumab | 13 | 0.000000 | Non PGx | Not High Risk |
| opticrom | 13 | 0.000000 | Non PGx | Not High Risk |
| oralieve | 13 | 0.000000 | Non PGx | Not High Risk |
| sorbaderm | 13 | 0.000000 | Non PGx | Not High Risk |
| synalar | 13 | 0.000000 | Non PGx | Not High Risk |
| atimos | 12 | 0.000000 | Non PGx | Not High Risk |
| balsalazide | 12 | 0.000000 | Non PGx | Not High Risk |
| cialis | 12 | 0.000000 | Non PGx | Not High Risk |
| dexafree | 12 | 0.000000 | Non PGx | Not High Risk |
| dextropropoxyphene | 12 | 0.000000 | Non PGx | Not High Risk |
| ethambutol | 12 | 0.000000 | Non PGx | Not High Risk |
| flavoxate | 12 | 0.000000 | Non PGx | Not High Risk |
| fluphenazine | 12 | 0.000000 | Non PGx | Not High Risk |
| gatalin | 12 | 0.000000 | Non PGx | Not High Risk |
| germoloids | 12 | 0.000000 | Non PGx | Not High Risk |
| half sinemet | 12 | 0.000000 | Non PGx | Not High Risk |
| hydrogen | 12 | 0.000000 | Non PGx | Not High Risk |
| lotriderm | 12 | 0.000000 | Non PGx | Not High Risk |
| mariosea | 12 | 0.000000 | Non PGx | Not High Risk |
| maxijul | 12 | 0.000000 | Non PGx | Not High Risk |
| mepacrine | 12 | 0.000000 | Non PGx | Not High Risk |
| nasofan | 12 | 0.000000 | Non PGx | Not High Risk |
| nizoral | 12 | 0.000000 | Non PGx | Not High Risk |
| rinatec | 12 | 0.000000 | Non PGx | Not High Risk |
| rituximab | 12 | 0.000000 | Non PGx | Not High Risk |
| sulpiride | 12 | 0.000000 | Non PGx | Not High Risk |
| tafluprost | 12 | 0.000000 | Non PGx | Not High Risk |
| zolmitriptan | 12 | 0.000000 | Non PGx | Not High Risk |
| oxcarbazepine | 11 | 0.000000 | PGx | Not High Risk |
| alprostadil | 11 | 0.000000 | Non PGx | Not High Risk |
| daktarin | 11 | 0.000000 | Non PGx | Not High Risk |
| entacapone | 11 | 0.000000 | Non PGx | Not High Risk |
| exorex | 11 | 0.000000 | Non PGx | Not High Risk |
| flivasorb | 11 | 0.000000 | Non PGx | Not High Risk |
| glucodrate | 11 | 0.000000 | Non PGx | Not High Risk |
| ixazomib | 11 | 0.000000 | Non PGx | Not High Risk |
| methylcellulose | 11 | 0.000000 | Non PGx | Not High Risk |
| nasonex | 11 | 0.000000 | Non PGx | Not High Risk |
| niraparib | 11 | 0.000000 | Non PGx | Not High Risk |
| omnitrope | 11 | 0.000000 | Non PGx | Not High Risk |
| phenoxybenzamine | 11 | 0.000000 | Non PGx | Not High Risk |
| pirfenidone | 11 | 0.000000 | Non PGx | Not High Risk |
| remedeine | 11 | 0.000000 | Non PGx | Not High Risk |
| repinex | 11 | 0.000000 | Non PGx | Not High Risk |
| robaxin | 11 | 0.000000 | Non PGx | Not High Risk |
| securon | 11 | 0.000000 | Non PGx | Not High Risk |
| senokot | 11 | 0.000000 | Non PGx | Not High Risk |
| sytron | 11 | 0.000000 | Non PGx | Not High Risk |
| abacavir | 10 | 0.000018 | PGx | Not High Risk |
| apomorphine | 10 | 0.000000 | Non PGx | Not High Risk |
| aztreonam | 10 | 0.000000 | Non PGx | Not High Risk |
| benzoyl peroxide | 10 | 0.000000 | Non PGx | Not High Risk |
| chlortalidone | 10 | 0.000000 | Non PGx | Not High Risk |
| duotrav | 10 | 0.000000 | Non PGx | Not High Risk |
| dymista | 10 | 0.000000 | Non PGx | Not High Risk |
| genotropin | 10 | 0.000000 | Non PGx | Not High Risk |
| ipinnia | 10 | 0.000000 | Non PGx | Not High Risk |
| kolanticon | 10 | 0.000000 | Non PGx | Not High Risk |
| liquigen | 10 | 0.000000 | Non PGx | Not High Risk |
| magnaspartate | 10 | 0.000000 | Non PGx | Not High Risk |
| modafinil | 10 | 0.000000 | Non PGx | Not High Risk |
| nutilis | 10 | 0.000000 | Non PGx | Not High Risk |
| ofloxacin | 10 | 0.000000 | Non PGx | Not High Risk |
| onbrez | 10 | 0.000000 | Non PGx | Not High Risk |
| perampanel | 10 | 0.000000 | Non PGx | Not High Risk |
| reboxetine | 10 | 0.000000 | Non PGx | Not High Risk |
| repaglinide | 10 | 0.000000 | Non PGx | Not High Risk |
| rifampicin | 10 | 0.000000 | Non PGx | Not High Risk |
| sandostatin | 10 | 0.000000 | Non PGx | Not High Risk |
| methadone | 9 | 0.000016 | PGx | Not High Risk |
| acamprosate | 9 | 0.000000 | Non PGx | Not High Risk |
| acebutolol | 9 | 0.000000 | Non PGx | Not High Risk |
| acrivastine | 9 | 0.000000 | Non PGx | Not High Risk |
| chlorambucil | 9 | 0.000000 | Non PGx | Not High Risk |
| chondroitin | 9 | 0.000000 | Non PGx | Not High Risk |
| ciclesonide | 9 | 0.000000 | Non PGx | Not High Risk |
| estraderm | 9 | 0.000000 | Non PGx | Not High Risk |
| ganciclovir | 9 | 0.000000 | Non PGx | Not High Risk |
| labetalol | 9 | 0.000000 | Non PGx | Not High Risk |
| levobunolol | 9 | 0.000000 | Non PGx | Not High Risk |
| mucogel | 9 | 0.000000 | Non PGx | Not High Risk |
| natecal | 9 | 0.000000 | Non PGx | Not High Risk |
| nyzamac | 9 | 0.000000 | Non PGx | Not High Risk |
| opicapone | 9 | 0.000000 | Non PGx | Not High Risk |
| promazine | 9 | 0.000000 | Non PGx | Not High Risk |
| travatan | 9 | 0.000000 | Non PGx | Not High Risk |
| venetoclax | 9 | 0.000000 | Non PGx | Not High Risk |
| bepanthen | 8 | 0.000000 | Non PGx | Not High Risk |
| bonefos | 8 | 0.000000 | Non PGx | Not High Risk |
| buspirone | 8 | 0.000000 | Non PGx | Not High Risk |
| butrans | 8 | 0.000000 | Non PGx | Not High Risk |
| cabergoline | 8 | 0.000000 | Non PGx | Not High Risk |
| castor | 8 | 0.000000 | Non PGx | Not High Risk |
| clioquinol | 8 | 0.000000 | Non PGx | Not High Risk |
| dexamfetamine | 8 | 0.000000 | Non PGx | Not High Risk |
| dicycloverine | 8 | 0.000000 | Non PGx | Not High Risk |
| dronedarone | 8 | 0.000000 | Non PGx | Not High Risk |
| ertapenem | 8 | 0.000000 | Non PGx | Not High Risk |
| etrivex | 8 | 0.000000 | Non PGx | Not High Risk |
| flaminal | 8 | 0.000000 | Non PGx | Not High Risk |
| forsteo | 8 | 0.000000 | Non PGx | Not High Risk |
| fulvestrant | 8 | 0.000000 | Non PGx | Not High Risk |
| gefitinib | 8 | 0.000000 | Non PGx | Not High Risk |
| lamictal | 8 | 0.000000 | Non PGx | Not High Risk |
| lamivudine | 8 | 0.000000 | Non PGx | Not High Risk |
| lenograstim | 8 | 0.000000 | Non PGx | Not High Risk |
| metrogel | 8 | 0.000000 | Non PGx | Not High Risk |
| mirapexin | 8 | 0.000000 | Non PGx | Not High Risk |
| nabilone | 8 | 0.000000 | Non PGx | Not High Risk |
| ocuvite | 8 | 0.000000 | Non PGx | Not High Risk |
| paliperidone | 8 | 0.000000 | Non PGx | Not High Risk |
| phenergan | 8 | 0.000000 | Non PGx | Not High Risk |
| tioconazole | 8 | 0.000000 | Non PGx | Not High Risk |
| tiopex | 8 | 0.000000 | Non PGx | Not High Risk |
| tostran | 8 | 0.000000 | Non PGx | Not High Risk |
| zaluron | 8 | 0.000000 | Non PGx | Not High Risk |
| azelaic | 7 | 0.000000 | Non PGx | Not High Risk |
| blephasol | 7 | 0.000000 | Non PGx | Not High Risk |
| combigan | 7 | 0.000000 | Non PGx | Not High Risk |
| daptomycin | 7 | 0.000000 | Non PGx | Not High Risk |
| demeclocycline | 7 | 0.000000 | Non PGx | Not High Risk |
| descovy | 7 | 0.000000 | Non PGx | Not High Risk |
| dexeryl | 7 | 0.000000 | Non PGx | Not High Risk |
| duoderm | 7 | 0.000000 | Non PGx | Not High Risk |
| eltrombopag | 7 | 0.000000 | Non PGx | Not High Risk |
| etoposide | 7 | 0.000000 | Non PGx | Not High Risk |
| fludroxycortide | 7 | 0.000000 | Non PGx | Not High Risk |
| hy-opti | 7 | 0.000000 | Non PGx | Not High Risk |
| jevity | 7 | 0.000000 | Non PGx | Not High Risk |
| kliovance | 7 | 0.000000 | Non PGx | Not High Risk |
| liothyronine | 7 | 0.000000 | Non PGx | Not High Risk |
| naltrexone | 7 | 0.000000 | Non PGx | Not High Risk |
| nexium | 7 | 0.000000 | Non PGx | Not High Risk |
| nilotinib | 7 | 0.000000 | Non PGx | Not High Risk |
| otex | 7 | 0.000000 | Non PGx | Not High Risk |
| prednisone | 7 | 0.000000 | Non PGx | Not High Risk |
| preservision | 7 | 0.000000 | Non PGx | Not High Risk |
| propiverine | 7 | 0.000000 | Non PGx | Not High Risk |
| scandishake | 7 | 0.000000 | Non PGx | Not High Risk |
| sofradex | 7 | 0.000000 | Non PGx | Not High Risk |
| stanek | 7 | 0.000000 | Non PGx | Not High Risk |
| truvada | 7 | 0.000000 | Non PGx | Not High Risk |
| ustekinumab | 7 | 0.000000 | Non PGx | Not High Risk |
| vedolizumab | 7 | 0.000000 | Non PGx | Not High Risk |
| acarbose | 6 | 0.000000 | Non PGx | Not High Risk |
| acidophilus | 6 | 0.000000 | Non PGx | Not High Risk |
| ampicillin | 6 | 0.000000 | Non PGx | Not High Risk |
| arachis | 6 | 0.000000 | Non PGx | Not High Risk |
| benepali | 6 | 0.000000 | Non PGx | Not High Risk |
| buccastem | 6 | 0.000000 | Non PGx | Not High Risk |
| choline | 6 | 0.000000 | Non PGx | Not High Risk |
| cyclopentolate | 6 | 0.000000 | Non PGx | Not High Risk |
| forxiga | 6 | 0.000000 | Non PGx | Not High Risk |
| frumil | 6 | 0.000000 | Non PGx | Not High Risk |
| germolene | 6 | 0.000000 | Non PGx | Not High Risk |
| granisetron | 6 | 0.000000 | Non PGx | Not High Risk |
| haleraid | 6 | 0.000000 | Non PGx | Not High Risk |
| hydromoor | 6 | 0.000000 | Non PGx | Not High Risk |
| lixisenatide | 6 | 0.000000 | Non PGx | Not High Risk |
| luventa | 6 | 0.000000 | Non PGx | Not High Risk |
| minocycline | 6 | 0.000000 | Non PGx | Not High Risk |
| mizolastine | 6 | 0.000000 | Non PGx | Not High Risk |
| monofer | 6 | 0.000000 | Non PGx | Not High Risk |
| olbas | 6 | 0.000000 | Non PGx | Not High Risk |
| optifibre | 6 | 0.000000 | Non PGx | Not High Risk |
| optrex | 6 | 0.000000 | Non PGx | Not High Risk |
| orabase | 6 | 0.000000 | Non PGx | Not High Risk |
| oxymetazoline | 6 | 0.000000 | Non PGx | Not High Risk |
| phenelzine | 6 | 0.000000 | Non PGx | Not High Risk |
| prolia | 6 | 0.000000 | Non PGx | Not High Risk |
| pseudoephedrine | 6 | 0.000000 | Non PGx | Not High Risk |
| renapro | 6 | 0.000000 | Non PGx | Not High Risk |
| stexerol | 6 | 0.000000 | Non PGx | Not High Risk |
| sucroferric | 6 | 0.000000 | Non PGx | Not High Risk |
| tolvaptan | 6 | 0.000000 | Non PGx | Not High Risk |
| trametinib | 6 | 0.000000 | Non PGx | Not High Risk |
| zafirlukast | 6 | 0.000000 | Non PGx | Not High Risk |
| adapalene | 5 | 0.000000 | Non PGx | Not High Risk |
| adex | 5 | 0.000000 | Non PGx | Not High Risk |
| afatinib | 5 | 0.000000 | Non PGx | Not High Risk |
| amias | 5 | 0.000000 | Non PGx | Not High Risk |
| atovaquone | 5 | 0.000000 | Non PGx | Not High Risk |
| benylin | 5 | 0.000000 | Non PGx | Not High Risk |
| caphosol | 5 | 0.000000 | Non PGx | Not High Risk |
| cefuroxime | 5 | 0.000000 | Non PGx | Not High Risk |
| danaparoid | 5 | 0.000000 | Non PGx | Not High Risk |
| dasatinib | 5 | 0.000000 | Non PGx | Not High Risk |
| deferasirox | 5 | 0.000000 | Non PGx | Not High Risk |
| earcalm | 5 | 0.000000 | Non PGx | Not High Risk |
| efracea | 5 | 0.000000 | Non PGx | Not High Risk |
| emulsiderm | 5 | 0.000000 | Non PGx | Not High Risk |
| femoston | 5 | 0.000000 | Non PGx | Not High Risk |
| gemfibrozil | 5 | 0.000000 | Non PGx | Not High Risk |
| glucotabs | 5 | 0.000000 | Non PGx | Not High Risk |
| golimumab | 5 | 0.000000 | Non PGx | Not High Risk |
| guaifenesin | 5 | 0.000000 | Non PGx | Not High Risk |
| hypafix | 5 | 0.000000 | Non PGx | Not High Risk |
| ikervis | 5 | 0.000000 | Non PGx | Not High Risk |
| iloprost | 5 | 0.000000 | Non PGx | Not High Risk |
| imiquimod | 5 | 0.000000 | Non PGx | Not High Risk |
| imodium | 5 | 0.000000 | Non PGx | Not High Risk |
| imuderm | 5 | 0.000000 | Non PGx | Not High Risk |
| iopidine | 5 | 0.000000 | Non PGx | Not High Risk |
| ketotifen | 5 | 0.000000 | Non PGx | Not High Risk |
| levocetirizine | 5 | 0.000000 | Non PGx | Not High Risk |
| lipitor | 5 | 0.000000 | Non PGx | Not High Risk |
| liquivisc | 5 | 0.000000 | Non PGx | Not High Risk |
| liskonium | 5 | 0.000000 | Non PGx | Not High Risk |
| magnaphate | 5 | 0.000000 | Non PGx | Not High Risk |
| metyrapone | 5 | 0.000000 | Non PGx | Not High Risk |
| minoxidil | 5 | 0.000000 | Non PGx | Not High Risk |
| monuril | 5 | 0.000000 | Non PGx | Not High Risk |
| moxisylyte | 5 | 0.000000 | Non PGx | Not High Risk |
| mucodyne | 5 | 0.000000 | Non PGx | Not High Risk |
| nateglinide | 5 | 0.000000 | Non PGx | Not High Risk |
| nitrolingual | 5 | 0.000000 | Non PGx | Not High Risk |
| norethisterone | 5 | 0.000000 | Non PGx | Not High Risk |
| nutraplus | 5 | 0.000000 | Non PGx | Not High Risk |
| nutricrem | 5 | 0.000000 | Non PGx | Not High Risk |
| octenisan | 5 | 0.000000 | Non PGx | Not High Risk |
| olodaterol | 5 | 0.000000 | Non PGx | Not High Risk |
| otrivine | 5 | 0.000000 | Non PGx | Not High Risk |
| pazopanib | 5 | 0.000000 | Non PGx | Not High Risk |
| permethrin | 5 | 0.000000 | Non PGx | Not High Risk |
| pneumococcal | 5 | 0.000000 | Non PGx | Not High Risk |
| psoriderm | 5 | 0.000000 | Non PGx | Not High Risk |
| qv | 5 | 0.000000 | Non PGx | Not High Risk |
| respi-clear | 5 | 0.000000 | Non PGx | Not High Risk |
| secukinumab | 5 | 0.000000 | Non PGx | Not High Risk |
| senset | 5 | 0.000000 | Non PGx | Not High Risk |
| siopel | 5 | 0.000000 | Non PGx | Not High Risk |
| sunitinib | 5 | 0.000000 | Non PGx | Not High Risk |
| sunsense | 5 | 0.000000 | Non PGx | Not High Risk |
| triclosan | 5 | 0.000000 | Non PGx | Not High Risk |
| triptorelin | 5 | 0.000000 | Non PGx | Not High Risk |
| voractiv | 5 | 0.000000 | Non PGx | Not High Risk |
| zantac | 5 | 0.000000 | Non PGx | Not High Risk |
| zostavax | 5 | 0.000000 | Non PGx | Not High Risk |
| alprazolam | 4 | 0.000000 | Non PGx | Not High Risk |
| aminoacridine | 4 | 0.000000 | Non PGx | Not High Risk |
| arjun | 4 | 0.000000 | Non PGx | Not High Risk |
| arthrotec | 4 | 0.000000 | Non PGx | Not High Risk |
| azilect | 4 | 0.000000 | Non PGx | Not High Risk |
| bactroban | 4 | 0.000000 | Non PGx | Not High Risk |
| betaxolol | 4 | 0.000000 | Non PGx | Not High Risk |
| bromfenac | 4 | 0.000000 | Non PGx | Not High Risk |
| cardicor | 4 | 0.000000 | Non PGx | Not High Risk |
| cetrimide | 4 | 0.000000 | Non PGx | Not High Risk |
| chloroquine | 4 | 0.000000 | Non PGx | Not High Risk |
| colazide | 4 | 0.000000 | Non PGx | Not High Risk |
| decapeptyl | 4 | 0.000000 | Non PGx | Not High Risk |
| depixol | 4 | 0.000000 | Non PGx | Not High Risk |
| disulfiram | 4 | 0.000000 | Non PGx | Not High Risk |
| elleste-duet | 4 | 0.000000 | Non PGx | Not High Risk |
| elleste-solo | 4 | 0.000000 | Non PGx | Not High Risk |
| eroset | 4 | 0.000000 | Non PGx | Not High Risk |
| estradot | 4 | 0.000000 | Non PGx | Not High Risk |
| everolimus | 4 | 0.000000 | Non PGx | Not High Risk |
| evolocumab | 4 | 0.000000 | Non PGx | Not High Risk |
| frebini | 4 | 0.000000 | Non PGx | Not High Risk |
| glucojuice | 4 | 0.000000 | Non PGx | Not High Risk |
| hydroxypropyl | 4 | 0.000000 | Non PGx | Not High Risk |
| istin | 4 | 0.000000 | Non PGx | Not High Risk |
| kalcipos-d | 4 | 0.000000 | Non PGx | Not High Risk |
| kentera | 4 | 0.000000 | Non PGx | Not High Risk |
| lubristil | 4 | 0.000000 | Non PGx | Not High Risk |
| maxalt | 4 | 0.000000 | Non PGx | Not High Risk |
| mebendazole | 4 | 0.000000 | Non PGx | Not High Risk |
| mepolizumab | 4 | 0.000000 | Non PGx | Not High Risk |
| meptazinol | 4 | 0.000000 | Non PGx | Not High Risk |
| metabet | 4 | 0.000000 | Non PGx | Not High Risk |
| nadolol | 4 | 0.000000 | Non PGx | Not High Risk |
| neorecormon injection | 4 | 0.000000 | Non PGx | Not High Risk |
| nimodipine | 4 | 0.000000 | Non PGx | Not High Risk |
| niquitin | 4 | 0.000000 | Non PGx | Not High Risk |
| nytol | 4 | 0.000000 | Non PGx | Not High Risk |
| orphenadrine | 4 | 0.000000 | Non PGx | Not High Risk |
| osteocaps | 4 | 0.000000 | Non PGx | Not High Risk |
| phytomenadione | 4 | 0.000000 | Non PGx | Not High Risk |
| piriton | 4 | 0.000000 | Non PGx | Not High Risk |
| polytar | 4 | 0.000000 | Non PGx | Not High Risk |
| premique | 4 | 0.000000 | Non PGx | Not High Risk |
| progynova | 4 | 0.000000 | Non PGx | Not High Risk |
| regorafenib | 4 | 0.000000 | Non PGx | Not High Risk |
| resp-ease | 4 | 0.000000 | Non PGx | Not High Risk |
| rhinocort | 4 | 0.000000 | Non PGx | Not High Risk |
| somatropin | 4 | 0.000000 | Non PGx | Not High Risk |
| soolantra | 4 | 0.000000 | Non PGx | Not High Risk |
| sterculia | 4 | 0.000000 | Non PGx | Not High Risk |
| tear-lac | 4 | 0.000000 | Non PGx | Not High Risk |
| temozolomide | 4 | 0.000000 | Non PGx | Not High Risk |
| tolbutamide | 4 | 0.000000 | Non PGx | Not High Risk |
| transvasin | 4 | 0.000000 | Non PGx | Not High Risk |
| trimetazidine | 4 | 0.000000 | Non PGx | Not High Risk |
| versatis | 4 | 0.000000 | Non PGx | Not High Risk |
| vigabatrin | 4 | 0.000000 | Non PGx | Not High Risk |
| xalacom | 4 | 0.000000 | Non PGx | Not High Risk |
| efavirenz | 3 | 0.000005 | PGx | Not High Risk |
| glibenclamide | 3 | 0.000005 | PGx | Not High Risk |
| glibenclamide | 3 | 0.000005 | PGx | Not High Risk |
| trimipramine | 3 | 0.000005 | PGx | Not High Risk |
| acalabrutinib | 3 | 0.000000 | Non PGx | Not High Risk |
| alirocumab | 3 | 0.000000 | Non PGx | Not High Risk |
| alitretinoin | 3 | 0.000000 | Non PGx | Not High Risk |
| amifampridine | 3 | 0.000000 | Non PGx | Not High Risk |
| baricitinib | 3 | 0.000000 | Non PGx | Not High Risk |
| benzocaine | 3 | 0.000000 | Non PGx | Not High Risk |
| betacap | 3 | 0.000000 | Non PGx | Not High Risk |
| betaquik | 3 | 0.000000 | Non PGx | Not High Risk |
| betmiga | 3 | 0.000000 | Non PGx | Not High Risk |
| blephaclean | 3 | 0.000000 | Non PGx | Not High Risk |
| botulinum | 3 | 0.000000 | Non PGx | Not High Risk |
| bromocriptine | 3 | 0.000000 | Non PGx | Not High Risk |
| bydureon | 3 | 0.000000 | Non PGx | Not High Risk |
| camcolit | 3 | 0.000000 | Non PGx | Not High Risk |
| cefaclor | 3 | 0.000000 | Non PGx | Not High Risk |
| cerumol | 3 | 0.000000 | Non PGx | Not High Risk |
| cetaphil | 3 | 0.000000 | Non PGx | Not High Risk |
| chemocare | 3 | 0.000000 | Non PGx | Not High Risk |
| cholestagel | 3 | 0.000000 | Non PGx | Not High Risk |
| clemastine | 3 | 0.000000 | Non PGx | Not High Risk |
| clofazimine | 3 | 0.000000 | Non PGx | Not High Risk |
| co-amoxiclav | 3 | 0.000000 | Non PGx | Not High Risk |
| co-careldopa | 3 | 0.000000 | Non PGx | Not High Risk |
| cocois | 3 | 0.000000 | Non PGx | Not High Risk |
| coro-nitro | 3 | 0.000000 | Non PGx | Not High Risk |
| cozaar | 3 | 0.000000 | Non PGx | Not High Risk |
| cranberry | 3 | 0.000000 | Non PGx | Not High Risk |
| dalacin | 3 | 0.000000 | Non PGx | Not High Risk |
| dermax | 3 | 0.000000 | Non PGx | Not High Risk |
| dimethyl fumarate | 3 | 0.000000 | Non PGx | Not High Risk |
| dimeticone | 3 | 0.000000 | Non PGx | Not High Risk |
| doxadura | 3 | 0.000000 | Non PGx | Not High Risk |
| driclor | 3 | 0.000000 | Non PGx | Not High Risk |
| duodopa | 3 | 0.000000 | Non PGx | Not High Risk |
| ebixa | 3 | 0.000000 | Non PGx | Not High Risk |
| elidel | 3 | 0.000000 | Non PGx | Not High Risk |
| enbrel | 3 | 0.000000 | Non PGx | Not High Risk |
| erlotinib | 3 | 0.000000 | Non PGx | Not High Risk |
| etravirine | 3 | 0.000000 | Non PGx | Not High Risk |
| exjade | 3 | 0.000000 | Non PGx | Not High Risk |
| exocin | 3 | 0.000000 | Non PGx | Not High Risk |
| exocream | 3 | 0.000000 | Non PGx | Not High Risk |
| fluocinonide | 3 | 0.000000 | Non PGx | Not High Risk |
| flurazepam | 3 | 0.000000 | Non PGx | Not High Risk |
| foradil | 3 | 0.000000 | Non PGx | Not High Risk |
| frangula | 3 | 0.000000 | Non PGx | Not High Risk |
| galfer | 3 | 0.000000 | Non PGx | Not High Risk |
| glecaprevir | 3 | 0.000000 | Non PGx | Not High Risk |
| granocyte | 3 | 0.000000 | Non PGx | Not High Risk |
| hiprex | 3 | 0.000000 | Non PGx | Not High Risk |
| hyetellose | 3 | 0.000000 | Non PGx | Not High Risk |
| iglu gel | 3 | 0.000000 | Non PGx | Not High Risk |
| immunoglobulin | 3 | 0.000000 | Non PGx | Not High Risk |
| indacaterol | 3 | 0.000000 | Non PGx | Not High Risk |
| ivermectin | 3 | 0.000000 | Non PGx | Not High Risk |
| linaclotide | 3 | 0.000000 | Non PGx | Not High Risk |
| loceryl | 3 | 0.000000 | Non PGx | Not High Risk |
| lonsurf | 3 | 0.000000 | Non PGx | Not High Risk |
| lyxumia | 3 | 0.000000 | Non PGx | Not High Risk |
| macitentan | 3 | 0.000000 | Non PGx | Not High Risk |
| mct | 3 | 0.000000 | Non PGx | Not High Risk |
| metosyn | 3 | 0.000000 | Non PGx | Not High Risk |
| monomax | 3 | 0.000000 | Non PGx | Not High Risk |
| nasacort | 3 | 0.000000 | Non PGx | Not High Risk |
| nasobec | 3 | 0.000000 | Non PGx | Not High Risk |
| neilmed | 3 | 0.000000 | Non PGx | Not High Risk |
| nevirapine | 3 | 0.000000 | Non PGx | Not High Risk |
| nystan | 3 | 0.000000 | Non PGx | Not High Risk |
| oftaquix | 3 | 0.000000 | Non PGx | Not High Risk |
| olopatadine | 3 | 0.000000 | Non PGx | Not High Risk |
| otezla | 3 | 0.000000 | Non PGx | Not High Risk |
| pabrinex | 3 | 0.000000 | Non PGx | Not High Risk |
| palbociclib | 3 | 0.000000 | Non PGx | Not High Risk |
| paramax | 3 | 0.000000 | Non PGx | Not High Risk |
| paroven | 3 | 0.000000 | Non PGx | Not High Risk |
| pembrolizumab | 3 | 0.000000 | Non PGx | Not High Risk |
| peristeen | 3 | 0.000000 | Non PGx | Not High Risk |
| pethidine | 3 | 0.000000 | Non PGx | Not High Risk |
| phosex | 3 | 0.000000 | Non PGx | Not High Risk |
| picolax | 3 | 0.000000 | Non PGx | Not High Risk |
| pindolol | 3 | 0.000000 | Non PGx | Not High Risk |
| praxilene | 3 | 0.000000 | Non PGx | Not High Risk |
| predsol | 3 | 0.000000 | Non PGx | Not High Risk |
| progesterone | 3 | 0.000000 | Non PGx | Not High Risk |
| propylthiouracil | 3 | 0.000000 | Non PGx | Not High Risk |
| rezolsta | 3 | 0.000000 | Non PGx | Not High Risk |
| ritonavir | 3 | 0.000000 | Non PGx | Not High Risk |
| sativex | 3 | 0.000000 | Non PGx | Not High Risk |
| solvazinc | 3 | 0.000000 | Non PGx | Not High Risk |
| sudafed | 3 | 0.000000 | Non PGx | Not High Risk |
| sustanon | 3 | 0.000000 | Non PGx | Not High Risk |
| timoptol | 3 | 0.000000 | Non PGx | Not High Risk |
| tofacitinib | 3 | 0.000000 | Non PGx | Not High Risk |
| tolcapone | 3 | 0.000000 | Non PGx | Not High Risk |
| tranylcypromine | 3 | 0.000000 | Non PGx | Not High Risk |
| triamterene | 3 | 0.000000 | Non PGx | Not High Risk |
| vemurafenib | 3 | 0.000000 | Non PGx | Not High Risk |
| xalatan | 3 | 0.000000 | Non PGx | Not High Risk |
| zemret | 3 | 0.000000 | Non PGx | Not High Risk |
| acipimox | 2 | 0.000000 | Non PGx | Not High Risk |
| alphaderm | 2 | 0.000000 | Non PGx | Not High Risk |
| alphagan | 2 | 0.000000 | Non PGx | Not High Risk |
| alu-cap | 2 | 0.000000 | Non PGx | Not High Risk |
| alzain | 2 | 0.000000 | Non PGx | Not High Risk |
| ambrisentan | 2 | 0.000000 | Non PGx | Not High Risk |
| amylmetacresol | 2 | 0.000000 | Non PGx | Not High Risk |
| anakinra | 2 | 0.000000 | Non PGx | Not High Risk |
| antazoline | 2 | 0.000000 | Non PGx | Not High Risk |
| apraclonidine | 2 | 0.000000 | Non PGx | Not High Risk |
| aviptadil | 2 | 0.000000 | Non PGx | Not High Risk |
| azacitidine | 2 | 0.000000 | Non PGx | Not High Risk |
| benperidol | 2 | 0.000000 | Non PGx | Not High Risk |
| benzylpenicillin | 2 | 0.000000 | Non PGx | Not High Risk |
| beta carotene | 2 | 0.000000 | Non PGx | Not High Risk |
| bezalip | 2 | 0.000000 | Non PGx | Not High Risk |
| bortezomib | 2 | 0.000000 | Non PGx | Not High Risk |
| brodalumab | 2 | 0.000000 | Non PGx | Not High Risk |
| budelin | 2 | 0.000000 | Non PGx | Not High Risk |
| budenofalk | 2 | 0.000000 | Non PGx | Not High Risk |
| bupivacaine | 2 | 0.000000 | Non PGx | Not High Risk |
| buttercup | 2 | 0.000000 | Non PGx | Not High Risk |
| cabozantinib | 2 | 0.000000 | Non PGx | Not High Risk |
| califig | 2 | 0.000000 | Non PGx | Not High Risk |
| casodex | 2 | 0.000000 | Non PGx | Not High Risk |
| clobaderm | 2 | 0.000000 | Non PGx | Not High Risk |
| co-beneldopa | 2 | 0.000000 | Non PGx | Not High Risk |
| coconut | 2 | 0.000000 | Non PGx | Not High Risk |
| colestid | 2 | 0.000000 | Non PGx | Not High Risk |
| credalast | 2 | 0.000000 | Non PGx | Not High Risk |
| crizotinib | 2 | 0.000000 | Non PGx | Not High Risk |
| crystacide | 2 | 0.000000 | Non PGx | Not High Risk |
| dalmane | 2 | 0.000000 | Non PGx | Not High Risk |
| dansac | 2 | 0.000000 | Non PGx | Not High Risk |
| daratumumab | 2 | 0.000000 | Non PGx | Not High Risk |
| darunavir | 2 | 0.000000 | Non PGx | Not High Risk |
| day and night nurse flu capsules | 2 | 0.000000 | Non PGx | Not High Risk |
| deferiprone | 2 | 0.000000 | Non PGx | Not High Risk |
| detrusitol | 2 | 0.000000 | Non PGx | Not High Risk |
| dextrogel | 2 | 0.000000 | Non PGx | Not High Risk |
| disopyramide | 2 | 0.000000 | Non PGx | Not High Risk |
| doxapram | 2 | 0.000000 | Non PGx | Not High Risk |
| dropodex | 2 | 0.000000 | Non PGx | Not High Risk |
| emollin | 2 | 0.000000 | Non PGx | Not High Risk |
| enopen | 2 | 0.000000 | Non PGx | Not High Risk |
| ethosuximide | 2 | 0.000000 | Non PGx | Not High Risk |
| factor VIII | 2 | 0.000000 | Non PGx | Not High Risk |
| famotidine | 2 | 0.000000 | Non PGx | Not High Risk |
| felbinac | 2 | 0.000000 | Non PGx | Not High Risk |
| femseven | 2 | 0.000000 | Non PGx | Not High Risk |
| fluanxol | 2 | 0.000000 | Non PGx | Not High Risk |
| fml eye drops | 2 | 0.000000 | Non PGx | Not High Risk |
| formflex | 2 | 0.000000 | Non PGx | Not High Risk |
| fosinopril | 2 | 0.000000 | Non PGx | Not High Risk |
| fragmin | 2 | 0.000000 | Non PGx | Not High Risk |
| frusol | 2 | 0.000000 | Non PGx | Not High Risk |
| gammanorm | 2 | 0.000000 | Non PGx | Not High Risk |
| gtn spray | 2 | 0.000000 | Non PGx | Not High Risk |
| hydrex | 2 | 0.000000 | Non PGx | Not High Risk |
| imigran | 2 | 0.000000 | Non PGx | Not High Risk |
| inflectra | 2 | 0.000000 | Non PGx | Not High Risk |
| innozide | 2 | 0.000000 | Non PGx | Not High Risk |
| interferon | 2 | 0.000000 | Non PGx | Not High Risk |
| isotretinoin | 2 | 0.000000 | Non PGx | Not High Risk |
| kaltostat | 2 | 0.000000 | Non PGx | Not High Risk |
| kaolin | 2 | 0.000000 | Non PGx | Not High Risk |
| kemadrin | 2 | 0.000000 | Non PGx | Not High Risk |
| kliofem | 2 | 0.000000 | Non PGx | Not High Risk |
| lemsip | 2 | 0.000000 | Non PGx | Not High Risk |
| locoid | 2 | 0.000000 | Non PGx | Not High Risk |
| lotemax | 2 | 0.000000 | Non PGx | Not High Risk |
| loteprednol | 2 | 0.000000 | Non PGx | Not High Risk |
| lubiprostone | 2 | 0.000000 | Non PGx | Not High Risk |
| mandanol | 2 | 0.000000 | Non PGx | Not High Risk |
| maviret | 2 | 0.000000 | Non PGx | Not High Risk |
| maxtrex | 2 | 0.000000 | Non PGx | Not High Risk |
| melolin | 2 | 0.000000 | Non PGx | Not High Risk |
| menthoderm | 2 | 0.000000 | Non PGx | Not High Risk |
| mestinon | 2 | 0.000000 | Non PGx | Not High Risk |
| metatone | 2 | 0.000000 | Non PGx | Not High Risk |
| myribase | 2 | 0.000000 | Non PGx | Not High Risk |
| nebilet | 2 | 0.000000 | Non PGx | Not High Risk |
| neurontin | 2 | 0.000000 | Non PGx | Not High Risk |
| norditropin | 2 | 0.000000 | Non PGx | Not High Risk |
| nystaform | 2 | 0.000000 | Non PGx | Not High Risk |
| octenilin | 2 | 0.000000 | Non PGx | Not High Risk |
| oestrogel | 2 | 0.000000 | Non PGx | Not High Risk |
| olaparib | 2 | 0.000000 | Non PGx | Not High Risk |
| olsalazine | 2 | 0.000000 | Non PGx | Not High Risk |
| oraldene | 2 | 0.000000 | Non PGx | Not High Risk |
| osmolite | 2 | 0.000000 | Non PGx | Not High Risk |
| phosphates | 2 | 0.000000 | Non PGx | Not High Risk |
| pimecrolimus | 2 | 0.000000 | Non PGx | Not High Risk |
| povitulle | 2 | 0.000000 | Non PGx | Not High Risk |
| pradaxa | 2 | 0.000000 | Non PGx | Not High Risk |
| privigen | 2 | 0.000000 | Non PGx | Not High Risk |
| procarbazine | 2 | 0.000000 | Non PGx | Not High Risk |
| rawel | 2 | 0.000000 | Non PGx | Not High Risk |
| reminyl | 2 | 0.000000 | Non PGx | Not High Risk |
| revaxis | 2 | 0.000000 | Non PGx | Not High Risk |
| ribociclib | 2 | 0.000000 | Non PGx | Not High Risk |
| rifabutin | 2 | 0.000000 | Non PGx | Not High Risk |
| romiplostim | 2 | 0.000000 | Non PGx | Not High Risk |
| salicylic acid | 2 | 0.000000 | Non PGx | Not High Risk |
| scarsil | 2 | 0.000000 | Non PGx | Not High Risk |
| selsun | 2 | 0.000000 | Non PGx | Not High Risk |
| sevikar | 2 | 0.000000 | Non PGx | Not High Risk |
| slow sodium mr tablet | 2 | 0.000000 | Non PGx | Not High Risk |
| solgar | 2 | 0.000000 | Non PGx | Not High Risk |
| somatuline | 2 | 0.000000 | Non PGx | Not High Risk |
| sorafenib | 2 | 0.000000 | Non PGx | Not High Risk |
| sorbsan | 2 | 0.000000 | Non PGx | Not High Risk |
| spiroco | 2 | 0.000000 | Non PGx | Not High Risk |
| suboxone | 2 | 0.000000 | Non PGx | Not High Risk |
| telfa | 2 | 0.000000 | Non PGx | Not High Risk |
| transiderm-nitro | 2 | 0.000000 | Non PGx | Not High Risk |
| transtec | 2 | 0.000000 | Non PGx | Not High Risk |
| ultraproct | 2 | 0.000000 | Non PGx | Not High Risk |
| uvistat | 2 | 0.000000 | Non PGx | Not High Risk |
| varivax | 2 | 0.000000 | Non PGx | Not High Risk |
| vera-til | 2 | 0.000000 | Non PGx | Not High Risk |
| zepatier | 2 | 0.000000 | Non PGx | Not High Risk |
| zidovudine | 2 | 0.000000 | Non PGx | Not High Risk |
| zonisamide | 2 | 0.000000 | Non PGx | Not High Risk |
| amikacin | 1 | 0.000002 | PGx | Not High Risk |
| fluvoxamine | 1 | 0.000002 | PGx | Not High Risk |
| pimozide | 1 | 0.000002 | PGx | Not High Risk |
| vortioxetine | 1 | 0.000002 | PGx | Not High Risk |
| accuretic | 1 | 0.000000 | Non PGx | Not High Risk |
| agomelatine | 1 | 0.000000 | Non PGx | Not High Risk |
| akynzeo | 1 | 0.000000 | Non PGx | Not High Risk |
| albumin | 1 | 0.000000 | Non PGx | Not High Risk |
| allergenics emollient cream | 1 | 0.000000 | Non PGx | Not High Risk |
| aluminium | 1 | 0.000000 | Non PGx | Not High Risk |
| arcoxia | 1 | 0.000000 | Non PGx | Not High Risk |
| arimidex | 1 | 0.000000 | Non PGx | Not High Risk |
| armour thyroid | 1 | 0.000000 | Non PGx | Not High Risk |
| avanafil | 1 | 0.000000 | Non PGx | Not High Risk |
| avoca | 1 | 0.000000 | Non PGx | Not High Risk |
| barkat | 1 | 0.000000 | Non PGx | Not High Risk |
| benzbromarone | 1 | 0.000000 | Non PGx | Not High Risk |
| berocca | 1 | 0.000000 | Non PGx | Not High Risk |
| betadine | 1 | 0.000000 | Non PGx | Not High Risk |
| bettamousse | 1 | 0.000000 | Non PGx | Not High Risk |
| bevacizumab | 1 | 0.000000 | Non PGx | Not High Risk |
| bilastine | 1 | 0.000000 | Non PGx | Not High Risk |
| bio-quinone | 1 | 0.000000 | Non PGx | Not High Risk |
| biotin | 1 | 0.000000 | Non PGx | Not High Risk |
| biotrol | 1 | 0.000000 | Non PGx | Not High Risk |
| bismuth | 1 | 0.000000 | Non PGx | Not High Risk |
| blephagel | 1 | 0.000000 | Non PGx | Not High Risk |
| bosutinib | 1 | 0.000000 | Non PGx | Not High Risk |
| bupropion | 1 | 0.000000 | Non PGx | Not High Risk |
| busulfan | 1 | 0.000000 | Non PGx | Not High Risk |
| calpol | 1 | 0.000000 | Non PGx | Not High Risk |
| carboplatin | 1 | 0.000000 | Non PGx | Not High Risk |
| cardene | 1 | 0.000000 | Non PGx | Not High Risk |
| cardura | 1 | 0.000000 | Non PGx | Not High Risk |
| care | 1 | 0.000000 | Non PGx | Not High Risk |
| cefotaxime | 1 | 0.000000 | Non PGx | Not High Risk |
| ceftriaxone | 1 | 0.000000 | Non PGx | Not High Risk |
| cellusan | 1 | 0.000000 | Non PGx | Not High Risk |
| certolizumab | 1 | 0.000000 | Non PGx | Not High Risk |
| champix | 1 | 0.000000 | Non PGx | Not High Risk |
| circadin | 1 | 0.000000 | Non PGx | Not High Risk |
| citrafleet | 1 | 0.000000 | Non PGx | Not High Risk |
| clexane | 1 | 0.000000 | Non PGx | Not High Risk |
| clinifast | 1 | 0.000000 | Non PGx | Not High Risk |
| clinipore | 1 | 0.000000 | Non PGx | Not High Risk |
| clinishield | 1 | 0.000000 | Non PGx | Not High Risk |
| clobavate | 1 | 0.000000 | Non PGx | Not High Risk |
| clopixol | 1 | 0.000000 | Non PGx | Not High Risk |
| clozaril | 1 | 0.000000 | Non PGx | Not High Risk |
| co-danthramer | 1 | 0.000000 | Non PGx | Not High Risk |
| colifoam | 1 | 0.000000 | Non PGx | Not High Risk |
| colofac | 1 | 0.000000 | Non PGx | Not High Risk |
| cometriq | 1 | 0.000000 | Non PGx | Not High Risk |
| co-proxamol | 1 | 0.000000 | Non PGx | Not High Risk |
| cosmofer | 1 | 0.000000 | Non PGx | Not High Risk |
| cosmopor | 1 | 0.000000 | Non PGx | Not High Risk |
| co-tenidone | 1 | 0.000000 | Non PGx | Not High Risk |
| co-trimoxazole | 1 | 0.000000 | Non PGx | Not High Risk |
| covonia | 1 | 0.000000 | Non PGx | Not High Risk |
| cuderm | 1 | 0.000000 | Non PGx | Not High Risk |
| curatoderm | 1 | 0.000000 | Non PGx | Not High Risk |
| cuvitru | 1 | 0.000000 | Non PGx | Not High Risk |
| cymbalta | 1 | 0.000000 | Non PGx | Not High Risk |
| cytamen | 1 | 0.000000 | Non PGx | Not High Risk |
| dalivit | 1 | 0.000000 | Non PGx | Not High Risk |
| dandrazol | 1 | 0.000000 | Non PGx | Not High Risk |
| denela | 1 | 0.000000 | Non PGx | Not High Risk |
| dermacolor | 1 | 0.000000 | Non PGx | Not High Risk |
| dermalo | 1 | 0.000000 | Non PGx | Not High Risk |
| desmotabs | 1 | 0.000000 | Non PGx | Not High Risk |
| desogestrel | 1 | 0.000000 | Non PGx | Not High Risk |
| dexpanthenol | 1 | 0.000000 | Non PGx | Not High Risk |
| dibrompropamidine | 1 | 0.000000 | Non PGx | Not High Risk |
| dificlir | 1 | 0.000000 | Non PGx | Not High Risk |
| diflucortolone | 1 | 0.000000 | Non PGx | Not High Risk |
| diprosone | 1 | 0.000000 | Non PGx | Not High Risk |
| dornase | 1 | 0.000000 | Non PGx | Not High Risk |
| doxorubicin | 1 | 0.000000 | Non PGx | Not High Risk |
| duac | 1 | 0.000000 | Non PGx | Not High Risk |
| duomed | 1 | 0.000000 | Non PGx | Not High Risk |
| dupilumab | 1 | 0.000000 | Non PGx | Not High Risk |
| dydrogesterone | 1 | 0.000000 | Non PGx | Not High Risk |
| echinacea | 1 | 0.000000 | Non PGx | Not High Risk |
| eflornithine | 1 | 0.000000 | Non PGx | Not High Risk |
| elemental 028 Extra liquid | 1 | 0.000000 | Non PGx | Not High Risk |
| eliquis | 1 | 0.000000 | Non PGx | Not High Risk |
| entocort | 1 | 0.000000 | Non PGx | Not High Risk |
| etelcalcetide | 1 | 0.000000 | Non PGx | Not High Risk |
| ethinylestradiol | 1 | 0.000000 | Non PGx | Not High Risk |
| eucalyptus | 1 | 0.000000 | Non PGx | Not High Risk |
| ezetrol | 1 | 0.000000 | Non PGx | Not High Risk |
| factor XIII injection | 1 | 0.000000 | Non PGx | Not High Risk |
| famciclovir | 1 | 0.000000 | Non PGx | Not High Risk |
| ferrograd | 1 | 0.000000 | Non PGx | Not High Risk |
| finacea | 1 | 0.000000 | Non PGx | Not High Risk |
| fluarix | 1 | 0.000000 | Non PGx | Not High Risk |
| flucytosine | 1 | 0.000000 | Non PGx | Not High Risk |
| flunarizine | 1 | 0.000000 | Non PGx | Not High Risk |
| flutamide | 1 | 0.000000 | Non PGx | Not High Risk |
| flutter | 1 | 0.000000 | Non PGx | Not High Risk |
| fobumix | 1 | 0.000000 | Non PGx | Not High Risk |
| folpik | 1 | 0.000000 | Non PGx | Not High Risk |
| fycompa | 1 | 0.000000 | Non PGx | Not High Risk |
| gamolenic | 1 | 0.000000 | Non PGx | Not High Risk |
| genvoya | 1 | 0.000000 | Non PGx | Not High Risk |
| glucagen | 1 | 0.000000 | Non PGx | Not High Risk |
| haliborange | 1 | 0.000000 | Non PGx | Not High Risk |
| healthaid | 1 | 0.000000 | Non PGx | Not High Risk |
| hepatitis | 1 | 0.000000 | Non PGx | Not High Risk |
| herceptin | 1 | 0.000000 | Non PGx | Not High Risk |
| hyacyst | 1 | 0.000000 | Non PGx | Not High Risk |
| hydra-neb | 1 | 0.000000 | Non PGx | Not High Risk |
| hydroframe | 1 | 0.000000 | Non PGx | Not High Risk |
| hydromorphone | 1 | 0.000000 | Non PGx | Not High Risk |
| hyfiber | 1 | 0.000000 | Non PGx | Not High Risk |
| idelalisib | 1 | 0.000000 | Non PGx | Not High Risk |
| imuvac | 1 | 0.000000 | Non PGx | Not High Risk |
| indivina | 1 | 0.000000 | Non PGx | Not High Risk |
| ingenol | 1 | 0.000000 | Non PGx | Not High Risk |
| invokana | 1 | 0.000000 | Non PGx | Not High Risk |
| isentress | 1 | 0.000000 | Non PGx | Not High Risk |
| isodur | 1 | 0.000000 | Non PGx | Not High Risk |
| ispagel | 1 | 0.000000 | Non PGx | Not High Risk |
| ixekizumab | 1 | 0.000000 | Non PGx | Not High Risk |
| kalms | 1 | 0.000000 | Non PGx | Not High Risk |
| kenzem | 1 | 0.000000 | Non PGx | Not High Risk |
| ketamine | 1 | 0.000000 | Non PGx | Not High Risk |
| lanacane | 1 | 0.000000 | Non PGx | Not High Risk |
| ledipasvir | 1 | 0.000000 | Non PGx | Not High Risk |
| lioresal | 1 | 0.000000 | Non PGx | Not High Risk |
| lipantil | 1 | 0.000000 | Non PGx | Not High Risk |
| liskonum | 1 | 0.000000 | Non PGx | Not High Risk |
| livial | 1 | 0.000000 | Non PGx | Not High Risk |
| lodoxamide | 1 | 0.000000 | Non PGx | Not High Risk |
| lofric | 1 | 0.000000 | Non PGx | Not High Risk |
| lucentis | 1 | 0.000000 | Non PGx | Not High Risk |
| medijel | 1 | 0.000000 | Non PGx | Not High Risk |
| mefenamic | 1 | 0.000000 | Non PGx | Not High Risk |
| melophthal | 1 | 0.000000 | Non PGx | Not High Risk |
| menitorix | 1 | 0.000000 | Non PGx | Not High Risk |
| mepitel | 1 | 0.000000 | Non PGx | Not High Risk |
| mepyramine | 1 | 0.000000 | Non PGx | Not High Risk |
| meritene | 1 | 0.000000 | Non PGx | Not High Risk |
| meropenem | 1 | 0.000000 | Non PGx | Not High Risk |
| metoject | 1 | 0.000000 | Non PGx | Not High Risk |
| midostaurin | 1 | 0.000000 | Non PGx | Not High Risk |
| migalastat | 1 | 0.000000 | Non PGx | Not High Risk |
| migramax | 1 | 0.000000 | Non PGx | Not High Risk |
| mintec | 1 | 0.000000 | Non PGx | Not High Risk |
| mirena | 1 | 0.000000 | Non PGx | Not High Risk |
| misoprostol | 1 | 0.000000 | Non PGx | Not High Risk |
| mitotane | 1 | 0.000000 | Non PGx | Not High Risk |
| moclobemide | 1 | 0.000000 | Non PGx | Not High Risk |
| modecate | 1 | 0.000000 | Non PGx | Not High Risk |
| molaxole | 1 | 0.000000 | Non PGx | Not High Risk |
| murine | 1 | 0.000000 | Non PGx | Not High Risk |
| mydrilate | 1 | 0.000000 | Non PGx | Not High Risk |
| myfenax | 1 | 0.000000 | Non PGx | Not High Risk |
| natamycin | 1 | 0.000000 | Non PGx | Not High Risk |
| naturcare | 1 | 0.000000 | Non PGx | Not High Risk |
| nerisone | 1 | 0.000000 | Non PGx | Not High Risk |
| neupro | 1 | 0.000000 | Non PGx | Not High Risk |
| nicotinamide | 1 | 0.000000 | Non PGx | Not High Risk |
| night nurse capsule | 1 | 0.000000 | Non PGx | Not High Risk |
| nitromin | 1 | 0.000000 | Non PGx | Not High Risk |
| noqdirna | 1 | 0.000000 | Non PGx | Not High Risk |
| noxafil | 1 | 0.000000 | Non PGx | Not High Risk |
| nuelin | 1 | 0.000000 | Non PGx | Not High Risk |
| nutricia | 1 | 0.000000 | Non PGx | Not High Risk |
| nutriflex | 1 | 0.000000 | Non PGx | Not High Risk |
| nutrizym | 1 | 0.000000 | Non PGx | Not High Risk |
| octagam | 1 | 0.000000 | Non PGx | Not High Risk |
| odefsey | 1 | 0.000000 | Non PGx | Not High Risk |
| olmetec | 1 | 0.000000 | Non PGx | Not High Risk |
| olumiant | 1 | 0.000000 | Non PGx | Not High Risk |
| opatanol | 1 | 0.000000 | Non PGx | Not High Risk |
| opticare | 1 | 0.000000 | Non PGx | Not High Risk |
| optiflo | 1 | 0.000000 | Non PGx | Not High Risk |
| optilast | 1 | 0.000000 | Non PGx | Not High Risk |
| oronac | 1 | 0.000000 | Non PGx | Not High Risk |
| oxactin | 1 | 0.000000 | Non PGx | Not High Risk |
| oxprenolol | 1 | 0.000000 | Non PGx | Not High Risk |
| pamsvax | 1 | 0.000000 | Non PGx | Not High Risk |
| panadol | 1 | 0.000000 | Non PGx | Not High Risk |
| penicillamine | 1 | 0.000000 | Non PGx | Not High Risk |
| peptamen | 1 | 0.000000 | Non PGx | Not High Risk |
| pericyazine | 1 | 0.000000 | Non PGx | Not High Risk |
| permitabs | 1 | 0.000000 | Non PGx | Not High Risk |
| pevaryl | 1 | 0.000000 | Non PGx | Not High Risk |
| phosphate | 1 | 0.000000 | Non PGx | Not High Risk |
| physiotens | 1 | 0.000000 | Non PGx | Not High Risk |
| pipexus | 1 | 0.000000 | Non PGx | Not High Risk |
| pirinase | 1 | 0.000000 | Non PGx | Not High Risk |
| polycal | 1 | 0.000000 | Non PGx | Not High Risk |
| portia | 1 | 0.000000 | Non PGx | Not High Risk |
| povidone | 1 | 0.000000 | Non PGx | Not High Risk |
| pregaday | 1 | 0.000000 | Non PGx | Not High Risk |
| pregnacare | 1 | 0.000000 | Non PGx | Not High Risk |
| proguanil | 1 | 0.000000 | Non PGx | Not High Risk |
| prontoderm | 1 | 0.000000 | Non PGx | Not High Risk |
| prosure | 1 | 0.000000 | Non PGx | Not High Risk |
| prothiaden | 1 | 0.000000 | Non PGx | Not High Risk |
| provera | 1 | 0.000000 | Non PGx | Not High Risk |
| provigil | 1 | 0.000000 | Non PGx | Not High Risk |
| prozac | 1 | 0.000000 | Non PGx | Not High Risk |
| quadrivalent | 1 | 0.000000 | Non PGx | Not High Risk |
| radian | 1 | 0.000000 | Non PGx | Not High Risk |
| ranexa | 1 | 0.000000 | Non PGx | Not High Risk |
| ranibizumab | 1 | 0.000000 | Non PGx | Not High Risk |
| rapitil | 1 | 0.000000 | Non PGx | Not High Risk |
| remegel | 1 | 0.000000 | Non PGx | Not High Risk |
| resonium | 1 | 0.000000 | Non PGx | Not High Risk |
| rhubarb extract 5% + salicylic acid 1% Oromucosal Liquid | 1 | 0.000000 | Non PGx | Not High Risk |
| ribavirin | 1 | 0.000000 | Non PGx | Not High Risk |
| rifater | 1 | 0.000000 | Non PGx | Not High Risk |
| rigevidon | 1 | 0.000000 | Non PGx | Not High Risk |
| rilpivirine | 1 | 0.000000 | Non PGx | Not High Risk |
| rimexolone | 1 | 0.000000 | Non PGx | Not High Risk |
| riociguat | 1 | 0.000000 | Non PGx | Not High Risk |
| rozex | 1 | 0.000000 | Non PGx | Not High Risk |
| rufinamide | 1 | 0.000000 | Non PGx | Not High Risk |
| saflutan | 1 | 0.000000 | Non PGx | Not High Risk |
| salatac | 1 | 0.000000 | Non PGx | Not High Risk |
| selenase | 1 | 0.000000 | Non PGx | Not High Risk |
| semaglutide | 1 | 0.000000 | Non PGx | Not High Risk |
| singulair | 1 | 0.000000 | Non PGx | Not High Risk |
| sinutab | 1 | 0.000000 | Non PGx | Not High Risk |
| slow-k | 1 | 0.000000 | Non PGx | Not High Risk |
| sodium aurothiomalate | 1 | 0.000000 | Non PGx | Not High Risk |
| soltel | 1 | 0.000000 | Non PGx | Not High Risk |
| sondate | 1 | 0.000000 | Non PGx | Not High Risk |
| sorbxtra | 1 | 0.000000 | Non PGx | Not High Risk |
| spasmonal | 1 | 0.000000 | Non PGx | Not High Risk |
| spatone | 1 | 0.000000 | Non PGx | Not High Risk |
| stribild | 1 | 0.000000 | Non PGx | Not High Risk |
| strivit | 1 | 0.000000 | Non PGx | Not High Risk |
| tamiflu | 1 | 0.000000 | Non PGx | Not High Risk |
| targaxan | 1 | 0.000000 | Non PGx | Not High Risk |
| taurolidine | 1 | 0.000000 | Non PGx | Not High Risk |
| temgesic | 1 | 0.000000 | Non PGx | Not High Risk |
| temocillin | 1 | 0.000000 | Non PGx | Not High Risk |
| tenormin | 1 | 0.000000 | Non PGx | Not High Risk |
| testogel | 1 | 0.000000 | Non PGx | Not High Risk |
| tiaprofenic | 1 | 0.000000 | Non PGx | Not High Risk |
| tigecycline | 1 | 0.000000 | Non PGx | Not High Risk |
| tolfenamic | 1 | 0.000000 | Non PGx | Not High Risk |
| tolnaftate | 1 | 0.000000 | Non PGx | Not High Risk |
| trangina | 1 | 0.000000 | Non PGx | Not High Risk |
| trastuzumab | 1 | 0.000000 | Non PGx | Not High Risk |
| trientine | 1 | 0.000000 | Non PGx | Not High Risk |
| trizivir | 1 | 0.000000 | Non PGx | Not High Risk |
| tropicamide | 1 | 0.000000 | Non PGx | Not High Risk |
| truxima | 1 | 0.000000 | Non PGx | Not High Risk |
| typhim | 1 | 0.000000 | Non PGx | Not High Risk |
| tyrozets | 1 | 0.000000 | Non PGx | Not High Risk |
| valerian | 1 | 0.000000 | Non PGx | Not High Risk |
| velphoro | 1 | 0.000000 | Non PGx | Not High Risk |
| venclyxto | 1 | 0.000000 | Non PGx | Not High Risk |
| venofer | 1 | 0.000000 | Non PGx | Not High Risk |
| vesicare | 1 | 0.000000 | Non PGx | Not High Risk |
| vimovo | 1 | 0.000000 | Non PGx | Not High Risk |
| vincristine | 1 | 0.000000 | Non PGx | Not High Risk |
| vinorelbine | 1 | 0.000000 | Non PGx | Not High Risk |
| xanax | 1 | 0.000000 | Non PGx | Not High Risk |
| zestoretic | 1 | 0.000000 | Non PGx | Not High Risk |
| zestril | 1 | 0.000000 | Non PGx | Not High Risk |
| zetuvit | 1 | 0.000000 | Non PGx | Not High Risk |
| zimovane | 1 | 0.000000 | Non PGx | Not High Risk |
| zonegran | 1 | 0.000000 | Non PGx | Not High Risk |

**Appendix Table A2 Test for collinearity between the candidate predictor variables.**

| **Length Stay** | | | |
| --- | --- | --- | --- |
| **Predictors** | **Variance Inflation Factor (VIF)** | **Increased Standard Error** | **Tolerance** |
| Age | 1.17 | 1.08 | 0.86 |
| Sex | 1.02 | 1.01 | 0.98 |
| PGx | 1.48 | 1.22 | 0.67 |
| Total number of medicines on admission | 1.62 | 1.27 | 0.62 |
| Frailty Status | 1.30 | 1.14 | 0.77 |
| Ethnicity | 1.02 | 1.01 | 0.98 |
| Emergency Admission Flag | 1.15 | 1.07 | 0.87 |
| Number of hospitalisations to date | 1.12 | 1.06 | 0.89 |
| **Admission Type** | | | |
| Age | 1.04 | 1.02 | 0.96 |
| Sex | 1.01 | 1.00 | 0.99 |
| PGx | 1.56 | 1.25 | 0.64 |
| Total number of medicines on admission | 1.62 | 1.27 | 0.62 |
| Frailty Status | 1.10 | 1.05 | 0.91 |
| Ethnicity | 1.02 | 1.01 | 0.98 |
| Number of hospitalisations to date | 1.06 | 1.03 | 0.94 |
| **Repeat hospitalisation** | | | |
| Age | 1.13 | 1.06 | 0.88 |
| Sex | 1.02 | 1.01 | 0.98 |
| PGx | 1.47 | 1.21 | 0.68 |
| Total number of medicines on admission | 1.56 | 1.25 | 0.64 |
| Frailty Status | 1.14 | 1.07 | 0.87 |
| Ethnicity | 1.01 | 1.01 | 0.99 |
| Number of hospitalisations to date | 1.03 | 1.02 | 0.97 |

**Appendix Table A3 All-subset variable selection of the number of predictors by determining the number of predictor variables with the lowest sum of squares**

**[Lowest Sum of Squares]**

| Number of variables in the subset | Residual sum of squares (RSS) |
| --- | --- |
| 1 | 18965314 |
| 2 | 17748658 |
| 3 | 17030164 |
| 4 | 16950783 |
| 5 | 16941841 |
| 6 | 16936111 |
| 7 | 16931857 |
| 8 | 16929490 |
| 9 | 16928277 |
| 10 | 16927250 |
| 11 | 16926305 |
| 12 | 16925965 |
| 13 | 16925629 |
| 14 | 16925515 |
| 15 | 16925515 |
| 16 | 16925434 |
| 17 | 16925372 |
| 18 | 16925250 |
| 19 | 16924033 |
| 20 | 16924026 |

**Appendix Table A4**

The equations resulting from multivariable prediction modelling to predict the probability of the outcome based on the other variables in the admission dataset. The whole dataset was analysed.

Equation 1 shows outcome variable: length of stay

Equation 2 shows outcome variable: unplanned hospital admission

Equation 3 shows outcome variable: repeat hospital admission after a planned hospital admission

Equation 4 shows outcome variable: repeat hospital admission after an unplanned hospital admission

In each case the intercept is the constant in the equation. Each of the predictor variables is associated with a coefficient that relates to the relative weight of the variable on the overall outcome in the context of all the other variables.

| ***Prediction model equation for length of stay (Equation 1)***  *Length of stay = 2.994672 + (‘Age band 65-74’ x 1.03) + (‘Age band=85+’ x 0.93) + (‘Sex= Male’ x1.01) + (‘Total number of medicines’ x 1.00) + (‘Number of medicines with PGx association’ x 0.98) + (‘Frailty status=High’ x 3.34) + (‘Frailty status=Intermediate’x2.02) + (‘Frailty status=Not calculated’x0.01) +(‘Ethnicity=Asian’ x 0.97) + (‘Ethnicity=Black’x1.25) + (‘Ethnicity=Mixed’x0.72) + (‘Ethnicity=Not Stated’x1.00) + (‘Ethnicity=Other’x0.90) + (‘Type of admission=Unplanned’x1.09) + (‘Number of hospitalisations’ x 0.98)* |
| --- |
| ***Prediction model equation for unplanned admission (Equation 2)***  *Unplanned admission = 2.745498 + (‘Age band 65-74’ x 0.71) + (‘Age band=85+’ x 2.79) + (‘Sex= Male’ x 0.86) + (‘Total number of medicines’ x 1.04) + (‘Number of medicines with PGx association’ x 0.95) + (‘Frailty status=High’ x 9.64) + (‘Frailty status=Intermediate’ x 2.84) + (‘Frailty status=Not calculated’ x 1.37) + (‘Ethnicity=Asian’ x 1.23) + (‘Ethnicity=Black’ x 1.32) + (‘Ethnicity=Mixed’ x 1.21) + (‘Ethnicity=Not Stated’ x 0.51) + (‘Ethnicity=Other’x0.82) + (‘Number of hospitalisations’ x 1.01)* |
| ***Prediction model equation for repeat hospitalisation after a planned admission (Equation 3)***  *Repeat hospitalisation after planned admission = (‘Age band 65-74’ x 1.01) + (‘Age band=85+’ x 1.33) + (‘Sex= Male’ x 1.02) + (‘Total number of medicines’ x 1.02) + (‘Number of medicines with PGx association’ x 0.99) + (‘Frailty status=High’ x 3.15) + (‘Frailty status=Intermediate’ x 1.73) + (‘Frailty status=Not calculated’ x 0.68) +(‘Ethnicity=Asian’ x 1.23) + (‘Ethnicity=Black’ x 1.43) + (‘Ethnicity=Mixed’ x 0.36) + (‘Ethnicity=Not Stated’ x 0.30) + (‘Ethnicity=Other’x0.53) + (‘Number of hospitalisations ’ x 1.01) -0.0768281* |
| ***Prediction model equation for repeat hospitalisation after an unplanned admission (Equation 4)***  *Subsequent unplanned admission after unplanned admission = (‘Age band 65-74’ x 1.09) + (‘Age band=85+’ x 0.98) + (‘Sex= Male’ x 0.99) + (‘Total number of medicines’ x 1.03) + (‘Number of medicines with PGx association’ x 1.01) + (‘Frailty status=High’ x 1.60) + (‘Frailty status=Intermediate’ x 1.26) + (‘Frailty status=Not calculated’ x 0.70) +(‘Ethnicity=Asian’ x 0.90) + (‘Ethnicity=Black’ x 0.79) + (‘Ethnicity=Mixed’ x 0.68) x (‘Ethnicity=Not Stated’ x 0.62) + (‘Ethnicity=Other’ x 0.98) + (‘Number of hospitalisations to date ’x 1.01) - 0.2733540* |

**Appendix Table A5**

The dataset was stratified into three distinct groups based on Frailty status: High Frailty (16,748 people), Intermediate Frailty and Low Frailty. The equations result from multivariable prediction modelling to predicts the probability of the outcome based on the other variables in the admission dataset. For each of the groups modelling was carried out resulting in

Equations 5,6,7 shows outcome variable: length of stay for each group

Equations 8,9,10 show outcome variable: unplanned hospital admission

Equations 11,12,13 shows outcome variable: repeat hospital admission after a planned hospital admission

Equations 14,15,16 shows outcome variable: repeat hospital admission after an unplanned hospital admission

In each case the intercept is the constant in the equation. Each of the predictor variables is associated with a coefficient that relates to the relative weight of the variable on the overall outcome in the context of all the other variables.

| ***Prediction model equation for length of stay with high frailty status (Equation 5)***  *Length of Stay in High Frailty Status= 3.2594273 + (‘Age band 65-74’ x 1.06) + (‘Age band=85+’ x 0.89) + (‘Sex= Male’ x 1.03) + (‘Total number of medicines x 1.00) + (‘Number of medicines with PGx association’ x 1.01) + (‘Ethnicity=Asian’ x 1.00) + (‘Ethnicity=Black’ x 1.16) + (‘Ethnicity=Mixed’ x 0.65) + (‘Ethnicity=Not Stated’ x 1.04) + (‘Ethnicity=Other’ x 0.98) + (‘Type of Admission=Unplanned’ x 0.91) (‘Number of hospitalisations’ x 0.98)* |
| --- |
| ***Prediction model equation for length of stay with intermediate frailty status (Equation 6)***  *Length of Stay in Intermediate Frailty Status= 2.5862797 + (‘Age band 65-74’ x 1.05) + (‘Age band=85+’ x 0.98) + (‘Sex= Male’ x 1.00) + (‘Total number of medicines x 0.99) + (‘Number of medicines with PGx association’ x 1.00) + (‘Ethnicity=Asian’ x 0.99) + (‘Ethnicity=Black’ x 1.43) + (‘Ethnicity=Mixed’ x 0.56) + (‘Ethnicity=Not Stated’ x 1.01) + (‘Ethnicity=Other’ x 0.84) + (‘Type of Admission=Unplanned’ x 1.03) (‘Number of hospitalisations’ x 0.98)* |
| ***Prediction model equation for length of stay with low frailty status (Equation 7)***  *Length of Stay in Low Frailty Status= 1.5088820 + (‘Age band 65-74’ x 1.00) + (‘Age band=85+’ x 0.99) + (‘Sex= Male’ x 1.01) + (‘Total number of medicines x 1.01) + (‘Number of medicines with PGx association’ x 1.00) + (‘Ethnicity=Asian’ x 0.88) + (‘Ethnicity=Black’ x 1.07) + (‘Ethnicity=Mixed’ x 1.08) x (‘Ethnicity=Not Stated’ x 0.99) + (‘Ethnicity=Other’ x 0.83) + (‘Type of Admission=Unplanned’ x 1.18) (‘Number of hospitalisations’ x 1.00* |
| ***Prediction model equation for unplanned admission with high frailty status (Equation 8)***  *Unplanned Admission in High Frailty Status= 2.696030 + (‘Age band 65-74’ x 0.57) + (‘Age band=85+’ x 3.09) + (‘Sex= Male’ x 0.65) + (‘Total number of medicines’ x 1.02) + (‘Number of medicines with PGx association’ x 0.90) + (‘Ethnicity=Asian’ x 0.66) x (‘Ethnicity=Black’ x 1.38) + (‘Ethnicity=Mixed’ x 75,749) x (‘Ethnicity=Not Stated’ x 0.25) + (‘Ethnicity=Other’ x 0.57) + (‘Number of hospitalisations to date ’ x 0.99)* |
| ***Prediction model equation for unplanned admission with intermediate frailty status (Equation 9)***  *Unplanned Admission in Intermediate Frailty Status= 1.509941 + (‘Age band 65-74’ x 0.65) + (‘Age band=85+’ x 3.02) + (‘Sex= Male’ x 0.90) + (‘Total number of medicines’ x 1.04) + (‘Number of medicines with PGx association’ x 0.94) + (‘Ethnicity=Asian’ x 1.23) + (‘Ethnicity=Black’ x 1.08) + (‘Ethnicity=Mixed’ x 2.34) x (‘Ethnicity=Not Stated’ x 0.54) + (‘Ethnicity=Other’ x 0.86) + (‘Number of hospitalisations to date ’x 1.01)* |
| ***Prediction model equation for unplanned admission with low frailty status (Equation 10)***  *Unplanned Admission in Low Frailty Status= 0.44966 + (‘Age band 65-74’ x 0.77) + (‘Age band=85+’ x 2.46) + (‘Sex= Male’ x 0.87) + (‘Total number of medicines’ x 1.05) + (‘Number of medicines with PGx association’ x 0.95) + (‘Ethnicity=Asian’ x 1.36) x (‘Ethnicity=Black’ x 1.45) + (‘Ethnicity=Mixed’ x 0.89) + (‘Ethnicity=Not Stated’ x 0.52) + (‘Ethnicity=Other’ x 0.76) + (‘Number of hospitalisations to date ’x 1.05)* |
| ***Prediction model equation for repeat hospital admission after an unplanned admission with high frailty status (Equation 11)***  *Subsequent unplanned after unplanned admission in High frailty status= (‘Age band 65-74’ x 1.22) + (‘Age band=85+’ x 0.90) + (‘Sex= Male’ x 0.93) + (‘Total number of medicines x 1.03) + (‘Number of medicines with PGx association’ x 1.02) + (‘Ethnicity=Asian’ x 0.87) x (‘Ethnicity=Black’ x 0.70) x (‘Ethnicity=Mixed’ x 0.30) x (‘Ethnicity=Not Stated’ x 0.75) x (‘Ethnicity=Other’ x 0.64) x (‘Number of hospitalisations to date ’ x 1.01) - 0.07986* |
| ***Prediction model equation for repeat hospital admission after an unplanned admission with intermediate frailty status (Equation 12)***  *Subsequent unplanned admission after unplanned admission in Intermediate Frailty Status= (‘Age band 65-74’ x 1.11) + (‘Age band=85+’ x 1.07) + (‘Sex= Male’ x 1.03) + (‘Total number of medicines x 1.04) + (‘Number of medicines with PGx association’ x 0.99) + (‘Ethnicity=Asian’ x 0.99) x (‘Ethnicity=Black’ x 0.81) x (‘Ethnicity=Mixed’ x 0.73) x (‘Ethnicity=Not Stated’ x 0.70) x (‘Ethnicity=Other’ x 1.40) x (‘Number of hospitalisations to date ’x 1.01) - 0.483170* |
| ***Prediction model equation for repeat hospital admission after an unplanned admission with low frailty status (Equation 13)***  *Subsequent unplanned admission after unplanned admission in Low Frailty Status= (‘Age band 65-74’ x 0.88) + (‘Age band=85+’ x 1.06) + (‘Sex= Male’ x 1.05) + (‘Total number of medicines x 1.03) + (‘Number of medicines with PGx association’ x 1.00) + (‘Ethnicity=Asian’ x 0.89) x (‘Ethnicity=Black’ x 1.04) x (‘Ethnicity=Mixed’ x 1.01) x (‘Ethnicity=Not Stated’ x 0.40) x (‘Ethnicity=Other’ x 0.90) x (‘Number of hospitalisations to date ’x 1.03) - 0.83375* |
| ***Prediction model equation for repeat hospital admission after a planned admission with high frailty status (Equation 14)***  *Subsequent unplanned and planned admission in High frailty status= 0.300102 + (‘Age band 65-74’ x 1.06) + (‘Age band=85+’ x 1.19) + (‘Sex= Male’ x 1.01) + (‘Total number of medicines’ x 1.02) + (‘Number of medicines with PGx association’ x 0.91) + (‘Ethnicity=Asian’ x 1.07) x (‘Ethnicity=Black’ x 0.90) x (‘Ethnicity=Mixed’ x 0.00) x (‘Ethnicity=Not Stated’ x 0.55) x (‘Ethnicity=Other’ x 0.00) x (‘Number of hospitalisations to date ’ x 1.00)* |
| ***Prediction model equation for repeat hospital admission after a planned admission with intermediate frailty status (Equation 15)***  *Subsequent unplanned admission and planned admission in Intermediate Frailty Status= (‘Age band 65-74’ x 1.09) + (‘Age band=85+’ x 1.68) + (‘Sex= Male’ x 0.99) + (‘Total number of medicines’ x 1.03) + (‘Number of medicines with PGx association’ x 0.97) + (‘Ethnicity=Asian’ x 1.00) x (‘Ethnicity=Black’ x 1.89) x (‘Ethnicity=Mixed’ x 0.00) x (‘Ethnicity=Not Stated’ x 0.30) x (‘Ethnicity=Other’ x 0.51) x (‘Number of hospitalisations to date ’x 1.02) - 0.985531* |
| ***Prediction model equation for repeat hospital admission after a planned admission with low frailty status (Equation 16)***  *Subsequent unplanned admission and planned admission in Low Frailty Status= (‘Age band 65-74’ x 0.91) + (‘Age band=85+’ x 1.19) + (‘Sex= Male’ x 1.12) + (‘Total number of medicines’ x 1.01) + (‘Number of medicines with PGx association’ x 1.03) + (‘Ethnicity=Asian’ x 1.10) x (‘Ethnicity=Black’ x 1.27) x (‘Ethnicity=Mixed’ x 0.46) x (‘Ethnicity=Not Stated’ x 0.33) x (‘Ethnicity=Other’ x 0.74) x (‘Number of hospitalisations to date ’x 1.04) - 1.500337* |
